# Assessment of soil heavy metal pollution and associated ecological risk of agriculture dominated mid-channel bars in a subtropical river basin

**DOI:** 10.1038/s41598-023-38058-0

**Published:** 2023-07-09

**Authors:** Md. Mofizul Hoque, Aznarul Islam, Abu Reza Md. Towfiqul Islam, Subodh Chandra Pal, Sadik Mahammad, Edris Alam

**Affiliations:** 1 Department of Geography, Aliah University, 17 Gora Chand Road, Kolkata, 700014 India; 2 Department of Disaster Management, Begum Rokeya University, Rangpur, Bangladesh; 3 Department of Development Studies, Daffodil International University, Dhaka, 1216 Bangladesh; 4 Department of Geography, The University of Burdwan, Purba Bardhaman, West Bengal 713104 India; 5 Faculty of Resilience, Rabdan Academy, 114646 Abu Dhabi, United Arab Emirates; 6 Department of Geography and Environmental Studies, University of Chittagong, Chittagong, 4331 Bangladesh

**Keywords:** Environmental sciences, Environmental social sciences, Hydrology

## Abstract

The elevated concentrations of heavy metals in soil considerably threaten ecological and human health. To this end, the present study assesses metals pollution and its threat to ecology from the mid-channel bar’s (*char*) agricultural soil in the Damodar River basin, India. For this, the contamination factor (CF), enrichment factor (EF), geoaccumulation index (I_geo_), pollution index, and ecological risk index (RI) were measured on 60 soil samples at 30 stations (2 from each station, i.e., surface and sub-surface) in different parts of the mid-channel bar. The CF and EF indicate that both levels of *char* soil have low contamination and hence portray a higher potential for future enrichment by heavy metals. Moreover, I_geo_ portrays that soil samples are uncontaminated to moderately contaminated. Further, pollution indices indicate that all the samples (both levels) are unpolluted with a mean of 0.062 for surface soils and 0.048 for sub-surface soils. Both levels of the *char* have a low potentiality for ecological risk with an average RI of 0.20 for the surface soils and 0.19 for the sub-surface soils. Moreover, Technique for order preference by similarity to ideal solution (TOPSIS) indicates that the sub-surface soils have lower pollution than the surface soils. The geostatistical modeling reveals that the simple kriging technique was estimated as the most appropriate interpolation model. The present investigation exhibits that reduced heavy metal pollution is due to the sandy nature of soils and frequent flooding. However, the limited pollution is revealed due to the intensive agricultural practices on riverine* chars*. Therefore, this would be helpful to regional planners, agricultural engineers, and stakeholders in a basin area.

## Introduction

Soil contamination by heavy metals (HM) is a serious environmental and human health problem in several non-industrialized and industrialized countries^1^ as they are considered the most serious pollutants^2^. Due to increasing geologic and human activities, and global economic development, HM-induced soil pollution is a common problem worldwide^3–5^. Soil pollution or soil-related problems have arisen as a great threat to human society as numerous HM like arsenic (As), copper (Cu), cadmium (Cd), lead (Pb) chromium (Cr), nickel (Ni), mercury (Hg) and zinc (Zn) are accumulated and threaten the environment and public health^6^. HM and metalloids contaminate about 5 million soil sites worldwide, with their concentrations exceeding their permissible limits^7^. Their high concentration leads to many risks to ecosystems and humans through the damage to food chain, food quality, and agricultural production. The HM concentration in soil has a vital role in soil fertility and nutrient status^8^. Some metals like Cu, Zn, and Se are crucial elements that play a pivotal role in the regular growth of plants and living organisms, but high concentrations can be toxic^8^. The natural sources of these HM in the environment are maintained through different processes of its natural cycle such as rock weathering and volcanic eruptions^9,10^. The natural concentration of HM in soil tends to remain low (non-toxic). However, anthropogenic activities change the basic characteristics of soil (e.g., texture, cation exchange capacity or CEC, pH, and bulk density) causing HM deposition in the soil leading to elevated pollution^4,11^. The major anthropogenic activities of HM pollution include mining, smelting, oil refining, pesticide production, petrochemical production, use of pesticides and fertilizers and raw sewage sludge, etc.^4,12–14^. Thus, the human-induced elevated concentration of pedo-chemical (PC) parameters in the soil deserves a scientific assessment for the restoration of soil health.

Nowadays, HM pollution assessments are crucial because people are worried about HM risk all over the world^2^. HM pollution in the soil affects crops, human and ecological health directly or indirectly due to its accumulation in the terrestrial and aquatic ecosystems through the food chain^8^. Higher HM concentration in the agricultural field leads to soil and water pollution as their concentration exceeds the threshold level which impacts crop health and crop production directly due to the effect of soil microbiological imbalance and decreased soil fertility^13^. It also affects aquatic biota indirectly because agricultural effluents are discharged into the aquatic ecosystems. In the last few decades, HM pollution has been a major concern all over the world as humans are much more aware of their health as well as their ecological health. HM pollution leads to various diseases such as blackfoot, gastric disorder, vomiting, skin irritation, mucous membranes, heart problems, leukemia, anemia, hypertension, cardiovascular disorders, asthma, bronchitis/emphysema, and other respiratory diseases, and even cancer^15,16^. Under the permissible limit of HM, some metals like Cu, Fe, Zn, and even Cr (III) are crucial for human and aquatic biota health^9^. However, some metals like Hg, As, Pb and Cd are non-essential biologically and are very toxic for the living organism^15^. Moreover, riverine ecological health not only depends on the quantity, quality, and timing of water flow (i.e., environmental flow) but also the assurance of controlled concentrations of the HM in the soil profiles^17^.

Recently, several research works have been done on the assessment of HM pollution all over the world^8,9,15,18^. Moreover, numerous research works have also been conducted regarding the contamination of HM in water and sediments and their ecological risk^14,19–25^. In this context, various approaches such as geochemical, ecological geochemistry as well as soil contamination indicators have been considered to convert the HM concentrations into a single index value. For example, the contamination factor (CF) has been used by several researchers to evaluate the intensity of the HM concentrations in soil^10,26,27^. Besides, enrichment factor (EF) has been used to describe the presence of trace elements in soil^28–30^. Similarly, ecological risk factor (Er) has been employed to examine the status of trace elements in the soil samples^10,27,31^. In addition, ecological risk (RI)^26,30,31^ and geoaccumulation (I_geo_) indices have also been used to determine the concentrations of HM in soil^26–28^. Several researchers have developed pollution index (PI)^32^ and pollution load index (PLI)^26,33,34^ to assess HM contamination and ecological risk globally. Similarly, numerous studies assessed the chemical properties of soil including organic, inorganic, or radioactive pollutants^35–37^. Moreover, the geophysical soil properties^37,38^ and biological soil properties such as organism or biodegradation process have also been studied by several researchers^1,39,40^.

In the context of the Damodar River Basin (DRB) in India, various anthropogenic activities (e.g. mining, industrial, and agricultural operations) and natural processes (rock weathering, mineralization, and atmospheric deposition) induce HM concentration in the basin areas including agricultural fields^17^. Moreover, a huge amount of HM is discharged into the rivers owing to the exponential growth of population, industrialization, urbanization, land use land cover change, and modern agricultural practices (irrigation and use of fertilizer and pesticide)^17,41^. These activities in the DRB lead to the accumulation of HM in the river resulting in water pollution and deterioration of the riverine ecology^17^.

The literature survey reflects that several studies focused on the contamination of HM in soil and water bodies. Besides, it is found that many research works account for the implementations of geostatistical models^18^ based on their highest accuracy on the distribution of point datasets. Moreover, few studies^42^ focused on the uses of the technique for order preference by similarity to ideal solution (TOPSIS) multi-criteria decision making (MCDM) technique to prioritize the best situation in the field of physical environments and human-induced activities. However, the assessment of HM pollution and its ecological risk from the agricultural field of mid-channel bar that goes under water during flood events has not yet been done globally in general and in the context of the DRB. The previous works on the DRB are mainly focused on the identification of HM pollution due to anthropogenic activities in the form of urbanization, industrialization and intensive agricultural practice. Thus, an assessment of surface and sub-surface soil pollution is still not undertaken in the context of the DRB. Therefore, it would be a novel attempt to address the spatiality of the HM pollution of surface and sub-surface soil of the agriculture-dominated *chars* of the DRB using an integrated approach involving geochemical, geospatial and geostatistical aspects.

Hence, the present study intends to analyze the PC parameters of collected soil samples and measure the HM contamination level in collected soils. We also measured the HM pollutants that are discharged into the river directly escalating ecological risks, and threatening human and aquatic biota health. The primary objectives of the present study are (1) to find out the spatial variation of soil quality parameters of the surface and sub-surface samples, and (2) to assess the heavy metals pollution in surface and sub-surface soils and their associated ecological risk. An integrated approach with geospatial and geostatistical techniques with semivariogram models and TOPSIS has been applied to assess the best-fit model for the spatial distribution of pedo-chemical parameters and prioritization of soil pollution zones. This study would help create a baseline assessment for the development of soil-ecological health and sustainable agricultural practice that may result in ensuring food security in a better way. This will strive to achieve the sustainable development goals (SDG-2: zero hunger, SDG 14: life below, and SDG 15: life on land).

## Materials and methods

### Study area

The DRB is one of the most important river basins in India in terms of natural resources and lies in the states of Jharkhand (73.7%) and West Bengal (26.3%)^43^. This funnel-shaped river basin has great importance to the socio-economic lives of millions of people as it contains numerous mineral resources, industries, mining activities, and agrarian practices^41,44,45^. On the other hand, it has also an agonizing side marked by colossal floods in the DRB^46,47^ along with man-induced environmental degradation^48^. It spreads an area of approximately 23370.98 km^2^ and extends from 84°30′ E to 88°15′ E longitude and 22°15′ to 24°30′ N latitude^41,43^. Moreover, the geographical extension of the selected *char* area lies between 23°33′ 41″ to 23°31′ 15″ N latitude and 87°10′ 45″ to 87°14′ 22″ E longitude. The present study area covers an area of approximately 4.19 km^2^ (3.42 km^2^ of one *char* and 0.77 km^2^ of another *char*).

The tropical rain-fed river Damodar and its basin area experience the southwest monsoonal regime^43^. The mean annual temperature in the basin area lies between 18° and 24 °C in winter and 29°–35 °C in summer with a mean annual rainfall of 100–200 cm. Based on the census of India^49^, about 17.25 million people live with a 738 km^2^ population density in the basin area. Major cities like Dhanbad, Bokaro, Durgapur, Asansol, and Bardhaman are located in the DRB^41^. Intensive agriculture is practiced in the basin area including its *chars*. The gross irrigation command area of this basin is 5690 km^2^ out of which 300 km^2^ of land in the upper basin is being irrigated and 3640 km^2^ of irrigation potential land is created by DVC dams in the basin area^50^. National Waterway 35, from *Krishak setu* (Bardhaman on State Highway No. 8) to the confluence with Hooghly River near Purba Basudebpur, 46% of land along the Damodar River is characterized as agricultural, and on average 58% of Bardhaman district total population belongs to the agricultural population. The agricultural effluents with numerous pollutants are discharged into the river at different points from the agricultural field of its course directly or indirectly, leading to an ecological risk for the river system.

### Sampling design and data collection

**Figure 1:**
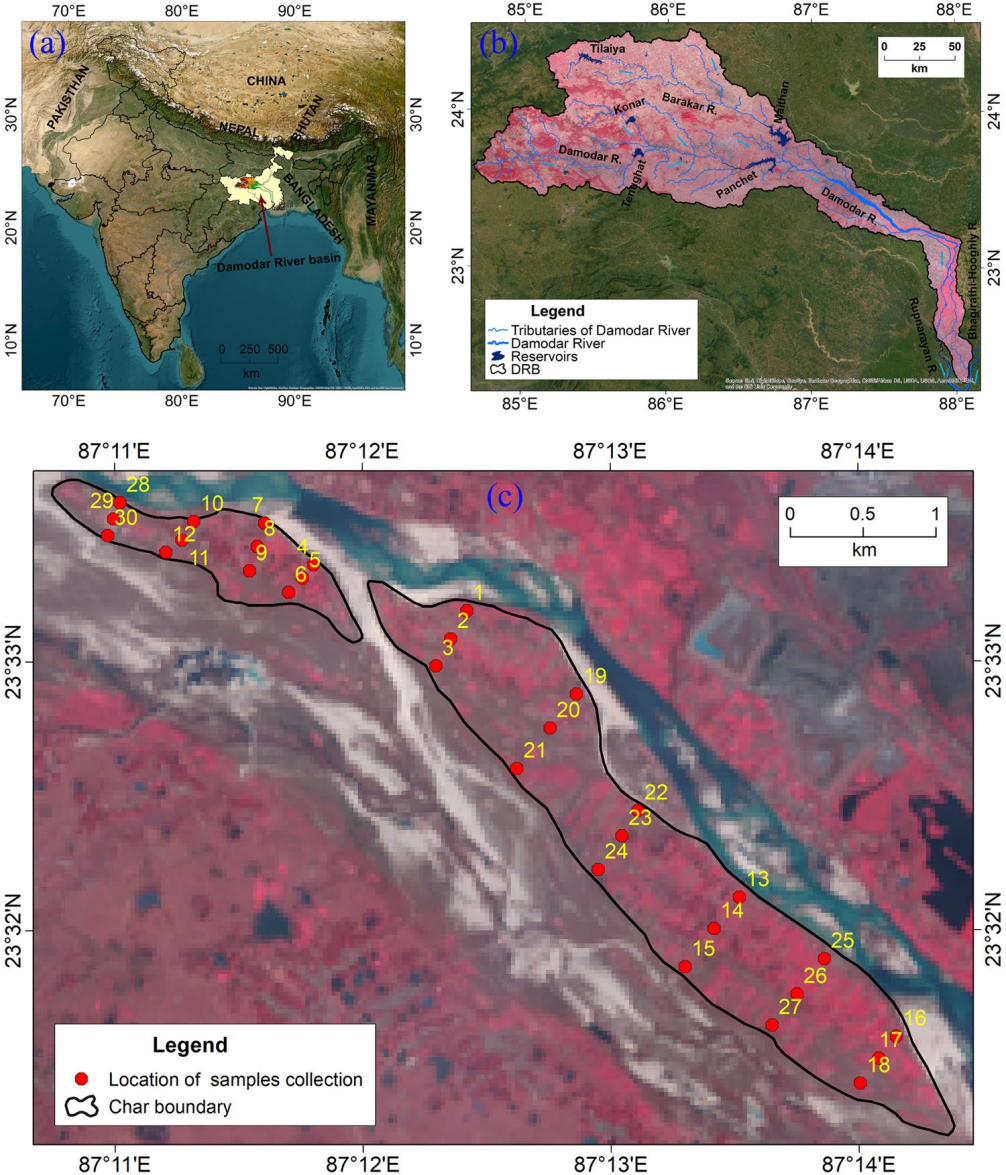
Location of the study *chars* and collection of soil samples. (**a**) location of the Damodar River Basin (DRB) in Eastern India, (**b**) drainage system of the DRB and location of dams and barrages under the Damodar Valley Corporation (DVC), (**c**) location of the study *chars *and soil samples (*Source:* prepared by the authors using ArcGIS software-version 10.2).

This study has been conducted based on the physico-chemical parameters of soils that have been collected from the mid-channel bars agricultural field of Damodar River. A total of 60 soils have been collected from the selected mid-channel bars (*chars*) agricultural at 30 different locations in 2021 (Fig. 1). Two soil samples were collected from each station i.e. one is from the surface level and another is from the sub-surface. As the *char* soils (fluvisols) are annually flood inundated, they do not exhibit mature soil profiles. Hence, soil profile depth has been considered as the criteria to segregate the surface and sub-surface soils. The depth of collected surface soils is 0–0.1 m and the sub-surface is 0.7–1 m (Table S1). Before sampling, the uppermost layer of soil has been removed for top-level soil collection, and for sub-surface soil collection, a digging bar (*Sabal*) is used to dig and collect soil. A quadrant pattern of the grid is used to collect soil samples in the study area^51^. Based on this method, a 1 m^2^ grid has been considered for taking soil samples and four samples from the surface soil in each location were collected and mixed well to prepare one soil surface sample. A similar approach was adopted for preparing the sub-surface soil samples. From each site, a well-mixed 500–600 g soil has been collected in an air-tight plastic polythene bag or pouch. After that, all the collected soils were corked, labeled carefully, and packed in another airtight bag. After collecting all the soils from the field, they were given for testing in the laboratory of Vivekananda Institute of Bio-Technology (Soil, Water and Manure Testing Laboratory) at Sri Ramkrishna Ashram in Nimpith, South 24-Parganas, West Bengal. The physico-chemical parameters such as pH, EC, OC, CEC, soil texture (sand, silt, and clay), soil moisture, and HM like Cu, Zn, Mn, Fe, and S were tested for each soil sample. We collected soils from *char* in pre-monsoon (February) because the concentration of pollution can be detected easily and efficiently. In other seasons (monsoon and post-monsoon), the concentration of pollution is low due to the monsoon and higher precipitation^52,53^. Moreover, we restricted our study only to pre-monsoon season because some parts of the *char* (mostly the north-western part) are low-lying and often become submerged under monsoon flood water.

### Pedo-chemical assessment and estimation of heavy metal pollution

**Figure 2:**
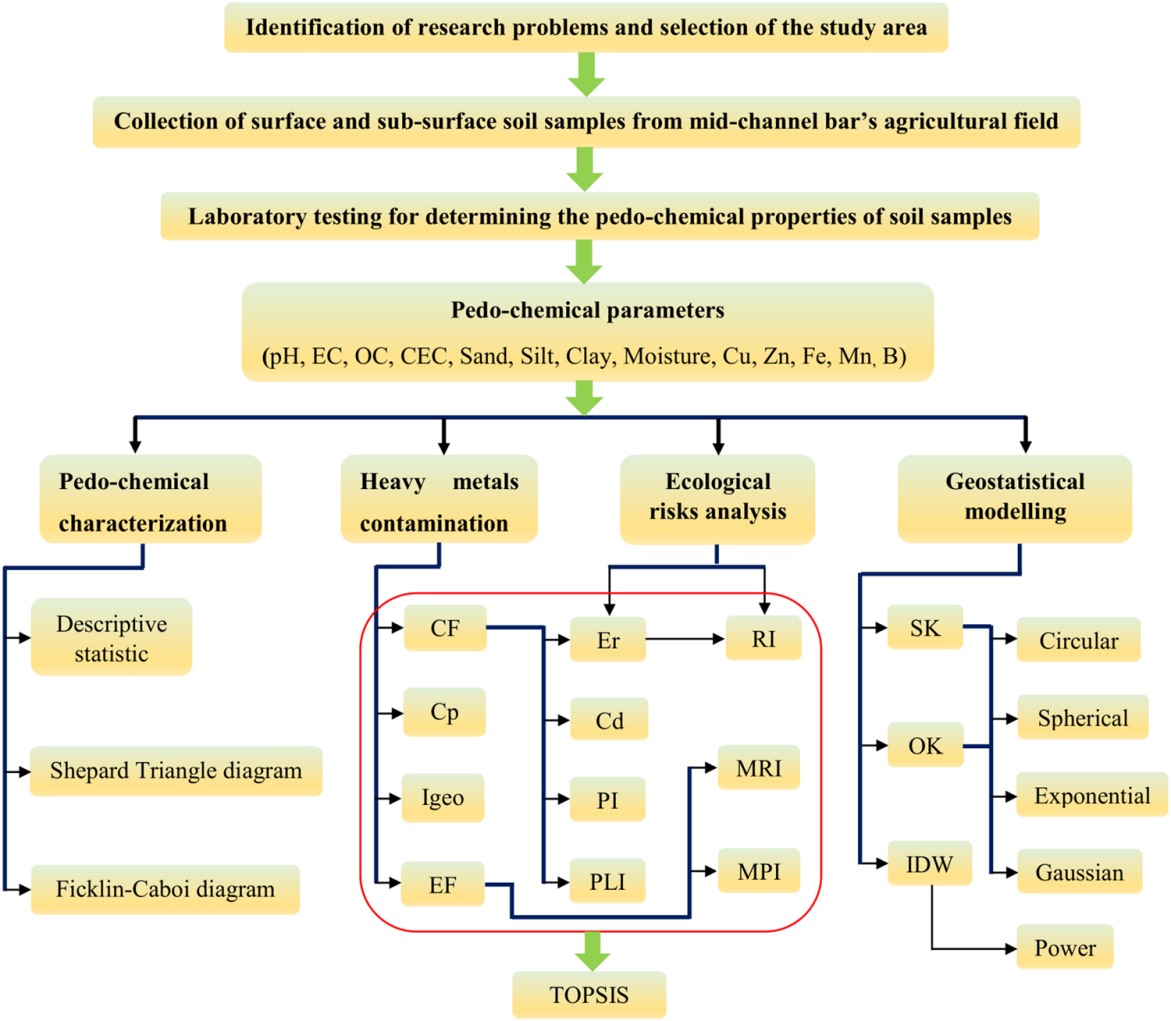
Flow chart of the methodology.

Pedo-chemical assessment has been executed using a robust methodology starting from the thematization, development of the research design for the collection of the data, and representation of the data with suitable geospatial and geo-statistical methods (Fig. 2).

#### Heavy metal pollution indices and ecological risk estimation

Several heavy metals contamination and ecological risk assessing methods such as contamination factor (CF), contamination degree (Cd), potential contamination index (Cp), enrichment factor (EF), geoaccumulation index (I_geo_), pollution index (PI), pollution load index (PLI), ecological risk index (RI), and potential ecological risk coefficient (Er) have been used for measuring HM pollution in soil and their risk to ecology from the studied soils samples. The CF, Cd, and Cp are used to assess HM contamination in soil^33,34,54^ while EF is used to measure HM enrichment in soil^55^ and to find out the potential sources of HM as the human or geogenic origin^10,33^. Similarly, I_geo_ is used to judge the level of metal accumulation in soil^10^. Moreover, PI and PLI are used to evaluate the pollution level in soil in a specific zone or site^33,56,57^. Finally, the ecological risk from HM is assessed through Er, RI and MRI to judge the sensitivity of different communities^30,54^. These methods employed in the present investigation are widely used for evaluating soil pollution and ecological risk from the PC parameters of soil. The details of these methods and algorithms are given in Table 1.

**Table 1:** Heavy metal pollution indices and ecological risk estimation.

Method	Equation	Description	Scale limit and classification system	References
Contamination factor	CF = ${ }\frac{{{\text{C}}_{{\text{o}}} }}{{{\text{C}}_{{\text{b}}} }}$	C_o_—observed metal concentration in soil; C_b_—background value	CF < 1 denotes low, 1 ≤ CF < 3 for moderate, 3 ≤ CF < 6 for considerable and 6 ≤ CF implies very high	^10,26,27^
Degree of contamination	Cd = $\sum\nolimits_{{{\text{i}} = 1}}^{{\text{n}}} {{\text{CF}}}$	CF—Contamination factor for metal*; n—*Number of HM	C deg < 8 represents low, 8 ≤ Cdeg < 16 for Moderate, 16 ≤ Cdeg < 32 for considerable, and 32 ≤ Cdeg for very high	^26,33^
Potential contamination index	Cp = ${ }\frac{{\left( {{\text{Metal}}} \right)_{{\text{sample Max}}} }}{{\left( {{\text{Metal}}} \right)_{{{\text{background}}}} }}$	Metal_sample Max_—maximum concentration of metal in soil; Metal _background_—background values of that metal	Cp < 1 for low, 1 < Cp < 3 for moderate and Cp > 3 for severe	^30,33^
Enrichment factor	EF = $\frac{{({\text{C}}_{{\text{x}}} /{\text{Fe}}){\text{sample}}}}{{\left( {{\text{C}}_{{\text{x}}} /{\text{Fe}}} \right){\text{background}}}}$	C_x sample_ and C_x background_—concentration of metal in soil and background environment; Fe _sample_ and Fe _background_—concentration of Fe in soil and the background environment	EF ≤ 1 for no enrichment, 1 < EF ≤ 3 for minor , 3 < EF ≤ 5 for moderate, 5 < EF ≤ 10 for moderately severe, 10 < EF ≤ 25 for severe enrichment, 25 < EF ≤ 50 for very severe and EF > 50 for extremely severe	^28–30^
Geoaccumulation index (I_geo_)	I_geo_ = log_2_ $\frac{{C_{n} }}{{1.5 \times B_{n} }}$	C_n_—metal concentration in soil sample; B_n_—background value	I_geo_ ≤ 0 represents practically uncontaminated, 0 ≤ I_geo_ < 1 for uncontaminated to moderately contaminated, 1 ≤ I_geo_ < 2 for moderately contaminated, 2 ≤ I_geo_ < 3 for moderately to heavily contaminated, 3 ≤ I_geo_ < 4 for heavily contaminated, 4 ≤ I_geo_ < 5 for heavily to extremely contaminated and 5 ≤ I_geo_ for extremely contaminated	^26–28^
Pollution index	PI = $\sqrt {\frac{{(CF_{average} )^{2} + (CF_{maximum} )^{2} }}{2}}$	CF_average_—average Contamination factor of metals; CF maximum—maximum contamination factor of metals	PI < 0.7 implies unpolluted, 0.7 < PI < 1 for slightly polluted, 1 < PI < 2 for modest polluted, 2 < PI < 3 for heavily polluted and PI > 3 for severely polluted	^32^
Modified pollution index	MPI = $\sqrt {\frac{{(EF_{average} )^{2} + (EF_{maximum} )^{2} }}{2}}$	EF_average_—average Enrichment factor of metals; CF_maximum_—maximum Enrichment factor of metals	MPI < 1 indicates unpolluted, 1 < MPI < 2 for slightly polluted, 2 < MPI < 3 for modest polluted, 3 < MPI < 5 for modest heavily polluted, 5 < MPI < 10 for heavily polluted and 10 < MPI for severely polluted	^32^
Pollution load index	PLI = $\sqrt[n]{CF1 \times CF2 \times CF3 \times \cdots CFn}$	CF—Contamination factor of each heavy metal; *n—*Number of HM	PLI < 1 for unpolluted, PLI = 1 for baseline level of pollution and PLI > 1 for polluted	^26,33^
Potential ecological risk coefficient	Er = Tr $\times$ CF	Tr—Toxic response factor; CF—Contamination factor	Er < 40 denotes low, 40 ≤ Er < 80 for moderate, 80 ≤ Er < 160 for considerable, 160 ≤ Er < 320 for high and 320 ≤ Er for very high	^10,27,31^
Ecological risk indices	RI = $\sum Tr \times CF$	Tr—Toxic response factor; CF—Contamination factor	RI < 150 for low, 150 ≤ RI < 300 for moderate, 300 ≤ RI < 600 for considerate, and RI > 600 for very high	^26,30,31^
Modified ecological risk indices	MRI = $\sum EF \times Tr$	EF—Enrichment factor; Tr—Toxic response factor	MRI < 150 for low, 150 ≤ MRI < 300 for moderate, 300 ≤ MRI < 600 for considerate, and MRI > 600 for very high	^30,32^

#### TOPSIS MCDM model

**Figure 3:**
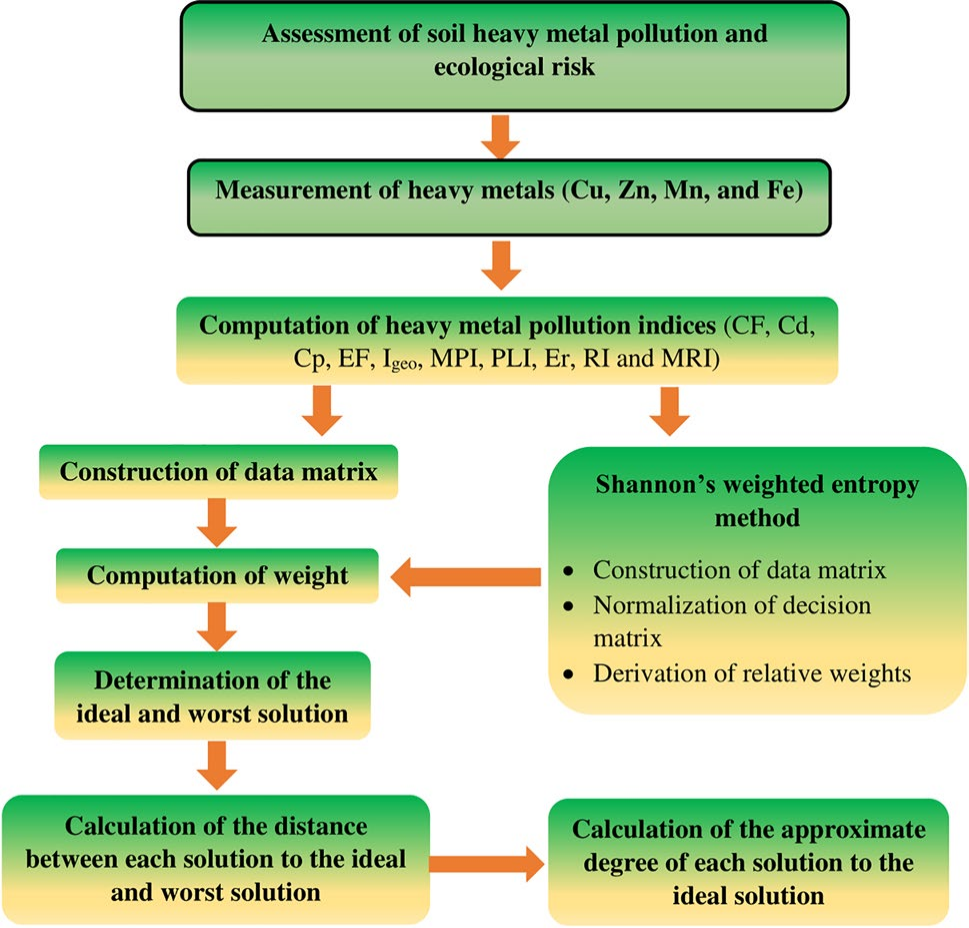
Flow chart of TOPSIS for prioritizing soil pollution zones.

Multi-criteria decision-making (MCDM) is a significant method to determine a problem that has multiple conflicting criteria in decision-making^58,59^. TOPSIS (Technique for Order of Preference by Similarity to Ideal Solution), an MCDM technique was invented by Hwang and Yoon in 1981. To calculate the optimum alternative, it accounts for both the shortest distance from the ideal solution and the longest distance from the anti-ideal solution at the same time^60^. Therefore, it is a useful technique to solve decision-making problems in the real world and is a widely used MCDM technique for prioritization purpose^61^. Hence, in the present investigation, the TOPSIS has been implemented to evaluate the priority of surface and sub-surface soil pollution. To avoid subjectivity, the weights of the variables used in the TOPSIS method, have been defined based on the Shannon entropy method^42,62^. Then, the calculated weights of different variables have been used in TOPSIS. The soil pollution and ecological risk assessing indices such as Cd, Cp, EF, I_geo_, MPI, PLI, Er, RI and MRI have been analyzed by TOPSIS for prioritizing the locations of the surface and sub-surface soil pollution for better management practices. The surface and sub-surface soil of the mid-channel bar is selected as the alternatives, and 16 variables related to soil pollution and ecological risk assessment are used as the criteria in the matrix table (Fig. 3).

#### Geostatistical analysis

The geostatistical model is used to analyze and predict the values related to spatial or spatiotemporal phenomena^63^. Geostatistical modeling has been used for the present study for error minimizing and finding the best-fit model to show the spatial distribution of variables. The best-fit model depends on the data nature and its spatial or spatiotemporal distribution. We applied geostatistical modeling for the spatial distribution of PC parameters and index values of soils. The interpolation methods like simple kriging (SK), ordinary kriging (OK), and inverse distance weighting (IDW) have been used for assessing the spatial distribution of PC parameters and index values of soils of the studied *chars*. The working principle and algorithms of SK, OK and IDW and best-fit model assessing tools like semi-variogram^18^ coupled with other statistical measures such as mean square error (MSE), mean error (ME), root mean square error (RMSE), root mean square standardized error (RMSSE), and average standard error (ASR) used in the present study are provided in supplementary (ST1). We used ArcGIS software (v. 10.2) for running the interpolation models and testing the best-fit ones.

## Results

### Pedo-chemical characterization

#### Soil textural distributions

**Figure 4:**
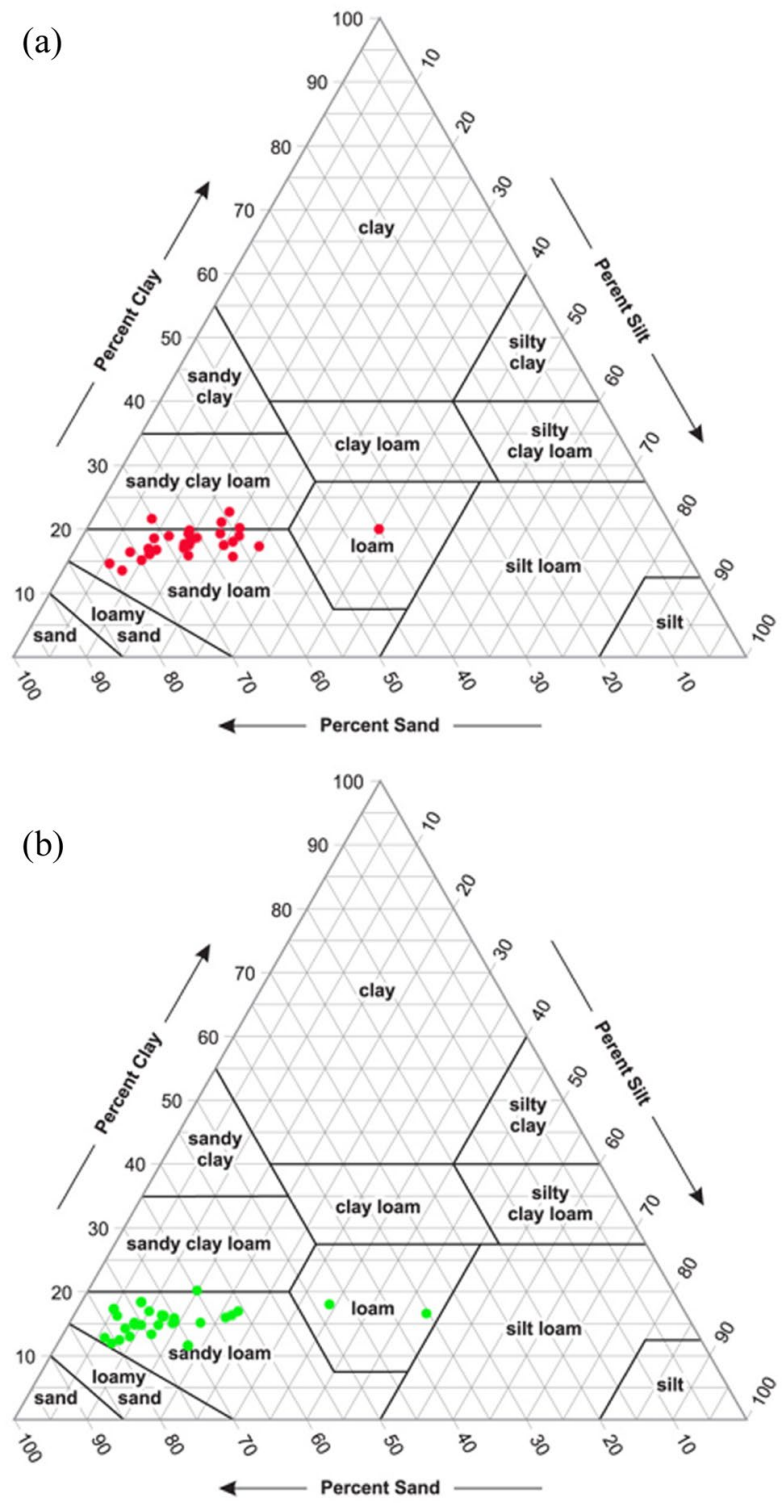
Classification of soil samples on Shepard triangle diagram. (**a**) surface soil, (**b**) sub-surface soil.

Textural analysis of soil is an important factor for studying the PC or HM pollution in soil because the concentration of PC elements, essential nutrients, and other living organisms depends on soil texture. The textural analysis exhibits that the majority of soils are mostly sandy and classified as fluvisols, a type of entisols developed by the fluvial deposits following annual flood events. In general, the sizes of soil particles affect the concentration of HM. The sizes of soil particles decrease with an increase in the concentration of HM in soil^64^. The surface soils depicted that 39.78–79.46% with a mean of 66.72% is sand, 6–40% with a mean of 15.48% is silt, and 13.38–22.56% with a mean of 17.94% is clay. For the sub-surface soils, 35.60–81.36% with a mean of 69.36%, 5–48% with a mean of 13.75% and 11.43–20.50% with a mean of 15.11% is sand, silt, and clay respectively (Table 2). Thus sub-surface soils are sandier than the surface. Furthermore, Shepard Triangle Diagram^65^ is used to classify the soils (Fig. 4a,b).

**Table 2:** Concentration of pedo-chemical parameters in the soil sample of the present study.

Parameters	Unit	A								B							
		Min	Max	Mean	SD	CV	Kurtosis	Skewness	K–S Test	Min	Max	Mean	SD	CV	Kurtosis	Skewness	K–S Test
pH		4.48	6.43	5.49	0.59	10.78	− 0.89	− 0.23	0.200	4.54	7.01	6.11	0.51	8.33	2.91	− 1.48	0.000
EC	dsm^−1^	0.05	0.69	0.2	0.15	76.3	3.55	1.8	0.038	0.03	0.37	0.11	0.08	77.04	4.91	2.15	0.001
OC	(%)	0.15	0.53	0.31	0.07	21.57	3.66	0.77	0.016	0.19	0.66	0.29	0.1	33.54	8.54	2.73	0.000
CEC	meq/100gm	2	5.44	3.99	0.83	20.77	0	− 0.39	0.173	2.52	11.48	6.77	2.26	33.4	− 0.48	0.19	0.200
Sand	(%)	39.78	79.46	66.72	7.84	11.75	3.56	− 1.19	0.200	35.6	81.36	69.36	9.66	13.93	4.48	− 1.79	0.039
Silt	(%)	6	40	15.48	6.76	43.64	4.76	1.64	0.163	5	48	13.75	8.87	64.49	7.48	2.48	0.001
Clay	(%)	13.38	22.56	17.94	2.06	11.51	0.15	0.15	0.200	11.43	20.5	15.11	1.93	12.76	1.12	0.71	0.009
Moisture	(%)	0.8	11.4	2.88	2.46	85.37	6.89	2.56	0.000	0.6	12	2.31	2.25	97.35	12.89	3.43	0.000
Cu	mg/kg	0.57	1.94	1	0.3	29.68	2.49	1.45	0.004	0.69	1.82	1.12	0.26	23.07	0.99	0.98	0.000
Zn	mg/kg	0.3	1.25	0.75	0.21	28.32	1.06	0.68	0.107	0.35	1.78	0.74	0.31	41.77	3.32	1.53	0.084
Fe	mg/kg	16.95	52.53	34.93	9.07	25.97	− 0.64	0.01	0.200	13.85	34.69	22.85	4.85	21.22	0.64	0.45	0.200
Mn	mg/kg	10.64	26	16.98	3.92	23.08	− 0.02	0.31	0.185	7.5	20.42	12.64	3.17	25.09	0.44	0.57	0.051
B	mg/kg	0.12	0.45	0.27	0.07	27.73	0.84	0.54	0.025	0.15	0.41	0.27	0.07	25.04	− 0.35	0.05	0.200

This diagram showed that 83.34, 13.33 and 3.33% of soils were sandy loam, sandy clay loam and loam of surface level. Besides, 90, 3.33 and 6.67% of soils were sandy loam, sandy clay loam and loam of sub-surface level respectively.

#### Soil pH and EC

**Figure 5:**
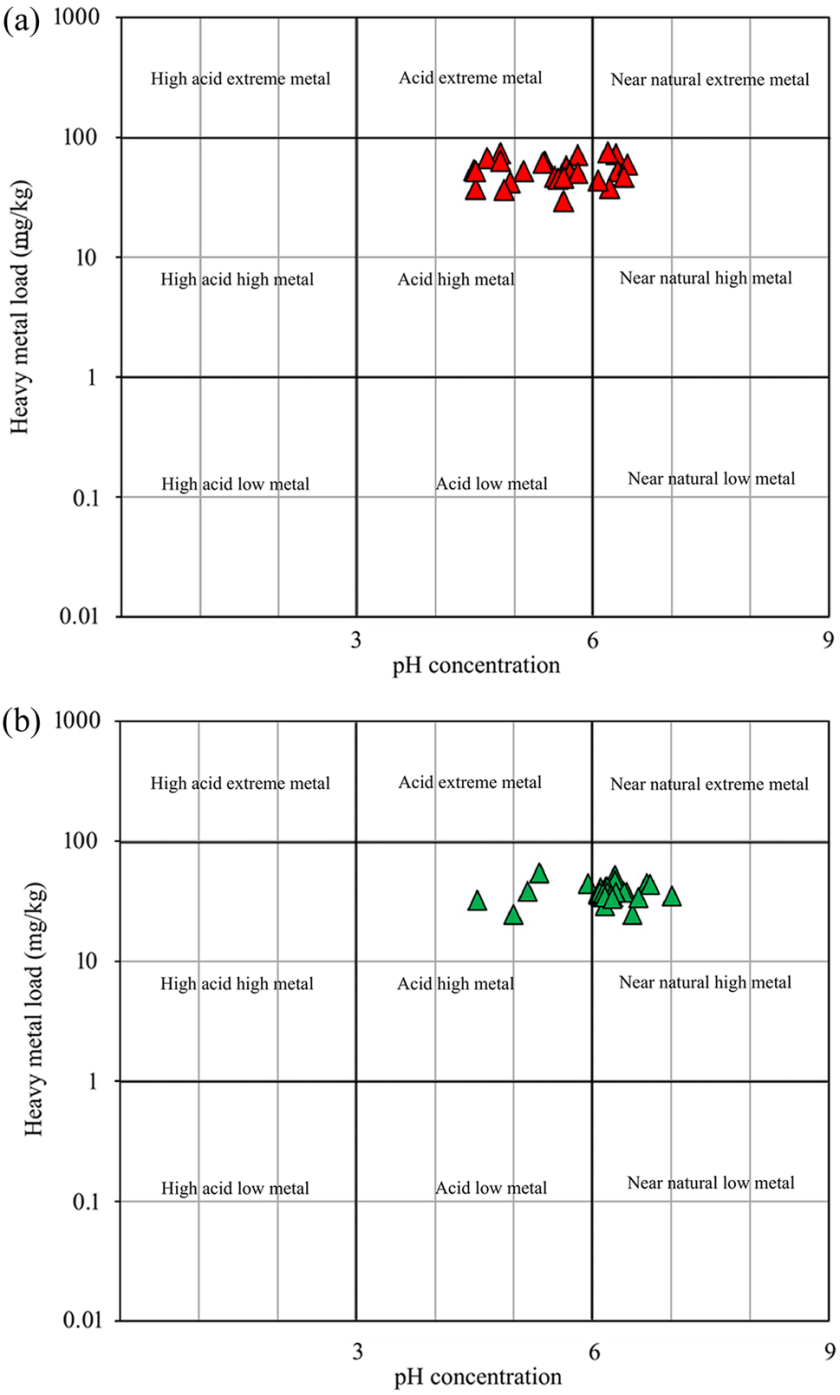
Classification of soil samples on Ficklin–Caboi diagram displaying HM load vs pH. (**a**) surface soils, (**b**) sub-surface soils.

**Figure 6:**
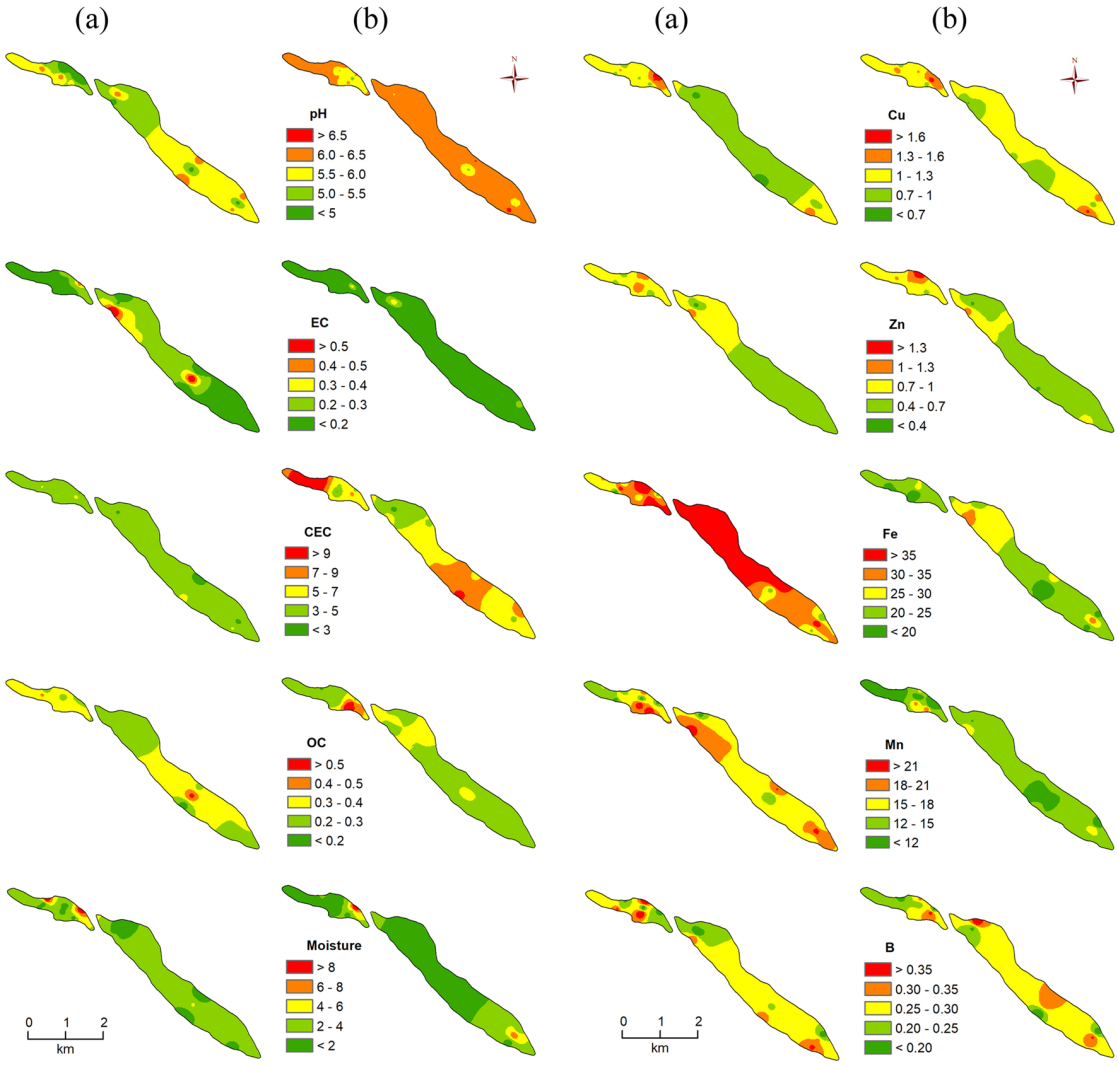
Spatial distribution of pedo-chemical (PC) parameters of the studied soils. (**a**) surface soil condition, (**b**) sub-surface soil condition (Note: units of the PC parameters are to be referred to Table 2).

The pH is an important chemical property of soil as it controls precipitation and adsorption which are the principal mechanisms of metal retention and affect metal dynamics in soil^64^. It plays a vital role in soil fertility as it controls the movement of HM and activates the macronutrients and trace elements in soil^26,66^. The present study found that surface soils were moderately acidic while the sub-surface was slightly acidic (surface pH = 5.49 and sub-surface pH = 6.11). The mean pH values of surface and sub-surface soils indicate that surface soil has nutrient deficiencies and toxicities (iron, manganese, and aluminum) and other elements (calcium, magnesium, nitrogen, phosphorous, and potassium) become less available for absorption by plants whereas sub-surface soil is an ideal soil condition for most plants. The pH of surface soils ranges from 4.48 to 6.43 while in the sub-surface soils, it ranges from 4.54 to 7.01 (Table 2). Based Ficklin–Caboi diagram, 76.67% of surface soil and 16.67% of sub-surface soils lie under acid high metal while 23.33% and 83.33% lie under near natural high metal respectively (Fig. 5a, b). Electrical conductivity (EC) is used to indicate the salinity of soil and water^67,68^. The present study found a low concentration of EC in the soils (0.05–0.69 dsm^−1^ with a mean of 0.20 dsm^−1^ for the surface soils and 0.03–0.37 dsm^−1^ with a mean value of 0.11 dsm^−1^ for the sub-surface soils (Table 2 and Fig. 6a,b). This result reveals that the surface soils are more saline than the sub-surface soils.

#### Organic carbon

Organic carbon (OC) is also a significant parameter of agricultural soil because it maintains the bio-availability of HM in soil^29^. A low concentration of OC in soil reduces the soil microbial diversity and biomass through decreasing mineralization. The concentration of OC (%) in the surface soils ranged from 0.15 to 0.53 while it varied from 0.19 to 0.66 for the sub-surface soils (Table 2 and Fig. 6a,b). These results indicate that the microbial diversity and biomass of sub-surface soils are lower than those of surface-level.

#### Soil moisture

Soil moisture also impacts the soil organisms and interacts with contaminants of soil. Hence, it affects the solubility of HM and the bioavailability of soil. It was found that the availability of soil moisture in surface soils ranged from 0.80 to 11.40% with a mean of 2.88% whereas it varied from 0.60 to 12% with a mean of 2.31% in the sub-surface soils (Table 2). Thus, the percentage of soil moisture concentration in the surface soils is more than the sub-surface level (Fig. 6a,b).

#### Cation exchange capacity

Soil CEC is a significant chemical property that reflects soil functions like structural stability, nutrient accessibility, pH, and reaction to fertilizers^64^. It is a measure of the soil's capability to bind interchangeable cations^64,66^. Our study findings indicate that the CEC (meq/100gm) of surface soils was found to be 2–5.44 with a mean of 3.99 while it was found to be 2.52–11.48 with a mean of 6.77 in the sub-surface soils (Table 2 and Fig. 6a,b). These results also indicate that the CEC of sub-surface soils has more ability to bind or hold the exchangeable cations than surface soils.

#### Heavy metals (Cu, Zn, Fe, Mn, B)

The concentration of HM is in the order of Fe > Mn > Cu > Zn > B based on the mean value of HM in both levels of soils (Fig. 6a,b). The concentration of Cu, Zn, Fe, Mn, and B in surface soils ranged from 0.57 to 1.94 mg/kg, 0.30 to 1.25 mg/kg, 16.95 to 52.53 mg/kg, 10.64 to 26 mg/kg and 0.12 to 0.45 mg/kg respectively whereas they ranged from 0.69 to 1.82 mg/kg, 0.35 to 1.78 mg/kg, 13.85 to 34.69 mg/kg, 7.50 to 20.42 mg/kg and 0.15 to 0.41 mg/kg in the sub-surface soils (Table 2). According to the mean values of the studied HM, the concentration of Zn, Fe, and Mn in surface soils is higher than in the sub-surface level. However, the concentration of Cu is lower than the sub-surface and interestingly the mean value of B is the same in both levels of samples. This study also found that the concentration of HM in the soils lies below the permissible limits (Cu = 100 mg/kg, Zn = 300 mg/kg, Fe = 2000 mg/kg, Mn = 50,000 mg/kg, and B = 30 mg/kg) based on Salem et al.^66^.

### Heavy metals contamination

#### Contamination factor

**Figure 7:**
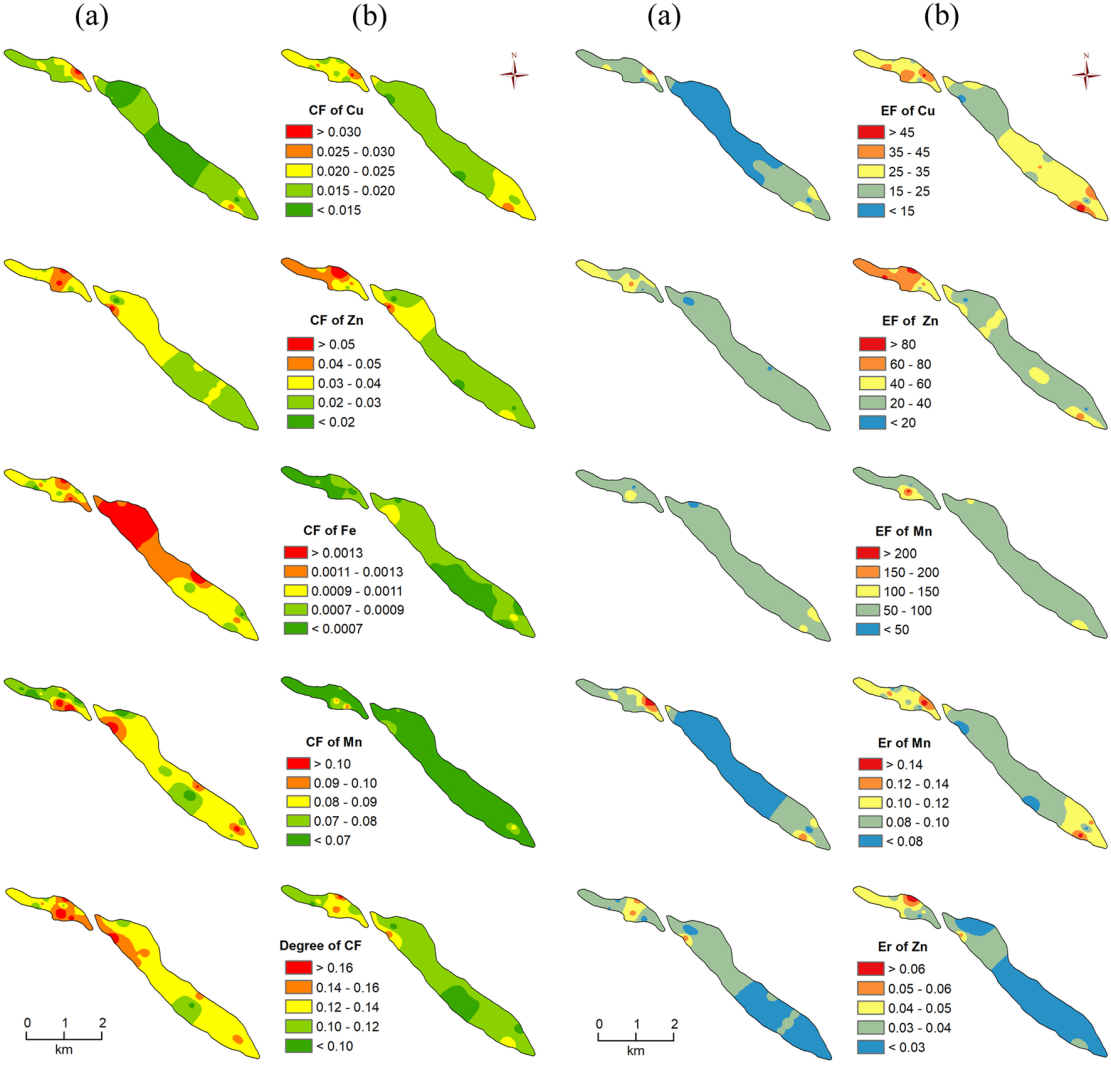
Spatial distribution of indices values of studied soils. (**a**) surface soil condition, (**b**) sub-surface soil condition.

It was found that the CF of Cu, Zn, Fe, and Mn in surface soils ranged from 0.010 to 0.034, 0.014 to 0.057, 0.00053 to 0.00164 and 0.051 to 0.124 respectively while it ranged from 0.012 to 0.032, 0.016 to 0.081, 0.00043 to 0.00108 and 0.036 to 0.098 in the sub-surface soils. Thus, these results show that all CF values lie below 1 (< 1), indicating that soils have a low level of contamination. From the obtained mean CF values of HM, it is observed that the CF values of Zn, Fe, and Mn of surface soils are higher than the sub-surface soils, however, the CF value of Cu is lower than the sub-surface. According to the average CF values of HM, they were ranked in the order of Mn > Zn > Cu > Fe in both levels of soils (Fig. 7a,b).

#### Contamination degree (Cd), and potential contamination index (Cp)

**Figure 8:**
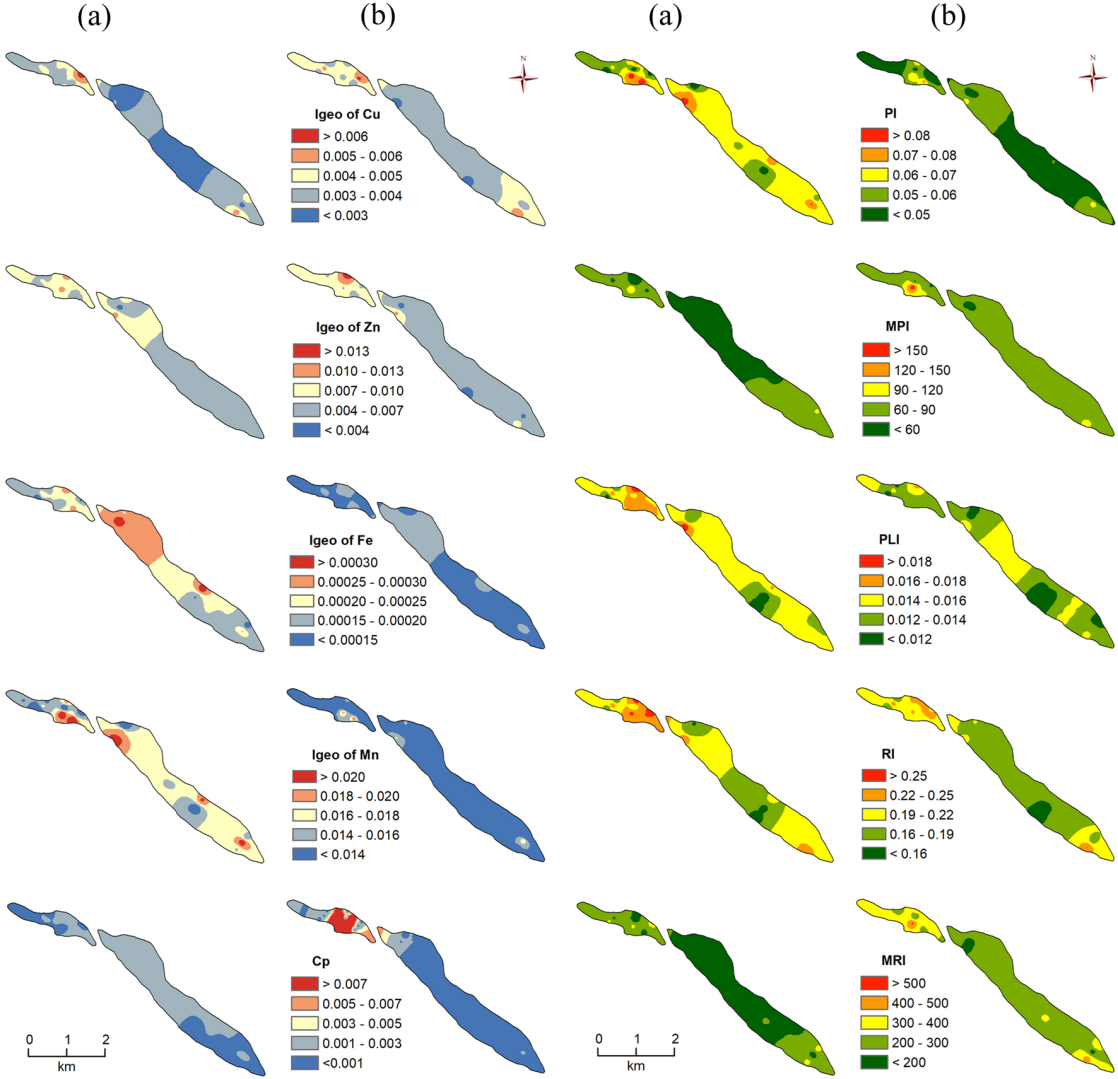
Spatial distribution of indices values of studied soils. (**a**) surface soil condition, (**b**) sub-surface soil condition.

The spatial distribution of Cd of surface soils was found to be 0.095–0.188 with a mean value of 0.134 whereas it was found to be 0.081–0.174 with a mean value of 0.114 in the sub-surface soils (Fig. 7a,b). The Cd of the studied HM is very low in all the soils. The study exhibits that all the Cd values from both level soils lie below 8C degrees which indicates that the metals have a low degree of contamination. The Cd values of surface soils are higher than sub-surface soils as compared to the range and mean of Cd values. The Cp values ranged from 0.00053 to 0.00164 in the surface soils whereas, in the sub-surface soils, they ranged from 0.00046 to 0.092 (Fig. 8a,b). The Cp values of the sub-surface soils are higher than the surface. All the calculated Cp values from both levels of soils lie below 1 which indicates that all the soils have low potential contamination of studied *chars*. All the Cp values are calculated from Fe as the highest concentration metal in soils but one Cp value is calculated from Mn (location ID. 9 of sub-surface soil) as it is the highest metal concentration. The Cp value of Zn is the highest among the other parameters.

#### Enrichment factor

The EF values of Cu, Zn, and Mn of surface soils were found to be 6.97–47.69, 8.48–67.09, and 41.39–142.48 respectively whereas they were found to be 11.89–51.03, 16.38–101.84 and 44.56–211.69 in the sub-surface soils (Fig. 7a,b). Based on the calculated EF mean values, it is observed that the EF values of sub-surface soils’ HM are higher than the surface soils. It is also observed that the EF value of Mn (in both levels) is high among the other investigated metals. According to their EF mean values, the investigated Cu, Zn, and Mn HM of surface soils are classified as severe, very severe, and extremely severe respectively. However, at the sub-surface level, they are classified as very severe, very severe, and extremely severe (Table 2). Moreover, they are ranked in the order of Mn > Zn > Cu for both levels of soil.

#### Geoaccumulation index (I_geo_)

The study found that the I_geo_ value of surface soils Cu, Zn, Fe, and Mn was 0.0020–0.0069, 0.0027–0.00114, 0.00011–0.00033 and 0.010–0.025 respectively whereas they were 0.0025–0.0065, 0.0032–0.00162, 0.00009–0.00022 and 0.007–0.020 in the sub-surface soils in the study. Therefore, these results show that all the calculated I_geo_ values lie below 1 (< 1) and Class 1 which indicates that soils are uncontaminated to moderately contaminated. The calculated I_geo_ mean values of HM reveal that the I_geo_ values of Zn, Fe, and Mn of sub-surface soils are lower than the sub-surface level, although the I_geo_ value of Cu is higher than the surface. Moreover, based on the mean I_geo_ values of HM, they were ranked in the order of Mn > Zn > Cu > Fe in both levels of soils (Fig. 8a,b).

#### Pollution index and pollution load index

In this study, the calculated pollution index values of surface soils ranged from 0. 040 to 0.093 with a mean value of 0.062 while it ranged from 0.031 to 0.073 in the sub-surface soils (Table S2 and Fig. 8a,b). Thus, based on these results, the pollution index values of surface soils are higher than the sub-surface. It indicates that the pollution level is also high in surface soils. Moreover, according to the classification of pollution index values, all the soils’ pollution index indices lie below 0.70 indicating the unpolluted nature of all the samples (from both levels). The PLI was found to be 0.11–0.020 in surface soils while 0.009–0.17 in the sub-surface soils (Fig. 8a,b). Hence, these results indicate that the pollution load of HM in surface soils is more than in the sub-surface.

### Ecological risk assessment

The ecological risk index (RI) shows that the RI values of surface soils ranged from 0.15 to 0.26 while it ranged from 0.14 to 0.25 in the sub-surface soils (Table S3). The RI values of surface soils are higher than the sub-surface. Hence, the ecological risk from the surface soil is higher than the sub-surface. Moreover, all the soils’ RI values lie under 150 indicating that all the samples from both levels have low ecological risk (Fig. 8a,b).

**Figure 9:**
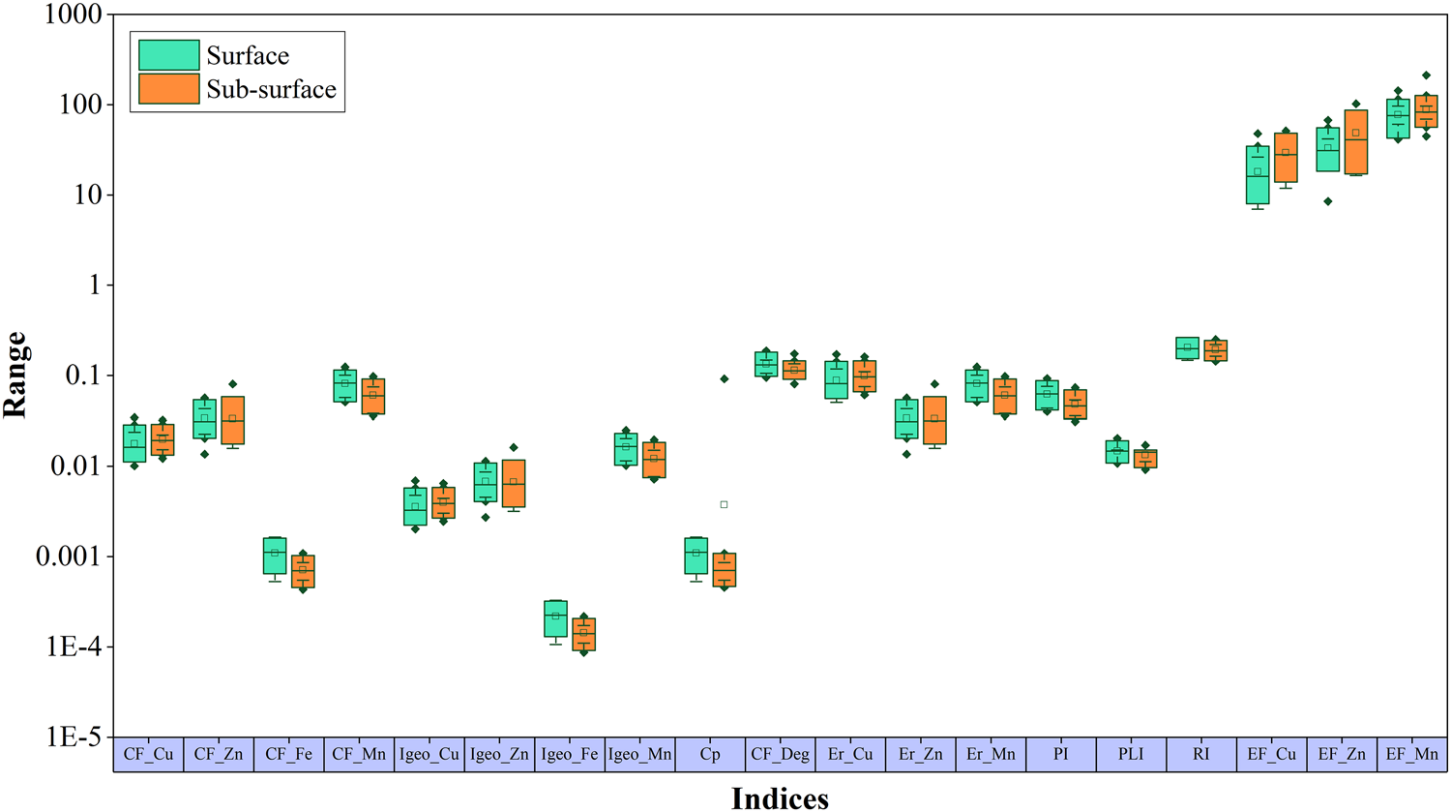
Box plot showing the variations in the heavy metal pollution indices.

The E^i^_r_ values of Cu, Zn, and Mn in the surface soils were 0.050–0.172, 0.014–0.057, and 0.051–0.124 respectively. However, they were found to be 0.061–0.161, 0.016–0.081 and 0.036–0.098 in the sub-surface soils (Table S3). The mean values of E^i^_r_ show that the E^i^_r_ values of Zn and Mn of surface soils are higher than the sub-surface but Cu is lower than the sub-surface soils. It is also found that E^i^_r_ value of Cu (in both levels) is high among the other investigated metals. The mean E^i^_r_ values of Cu, Zn, and Mn, E^i^_r_ values of each HM in all the soils from both levels were less than 40 and classified as low potential ecological risk. Therefore, the studied HM in soils has a low potentiality for ecological risk. Moreover, they are ranked as Cu > Zn > Mn for both levels of soil (Fig. 9).

### Prioritization of surface and sub-surface soil using TOPSIS

**Figure 10:**
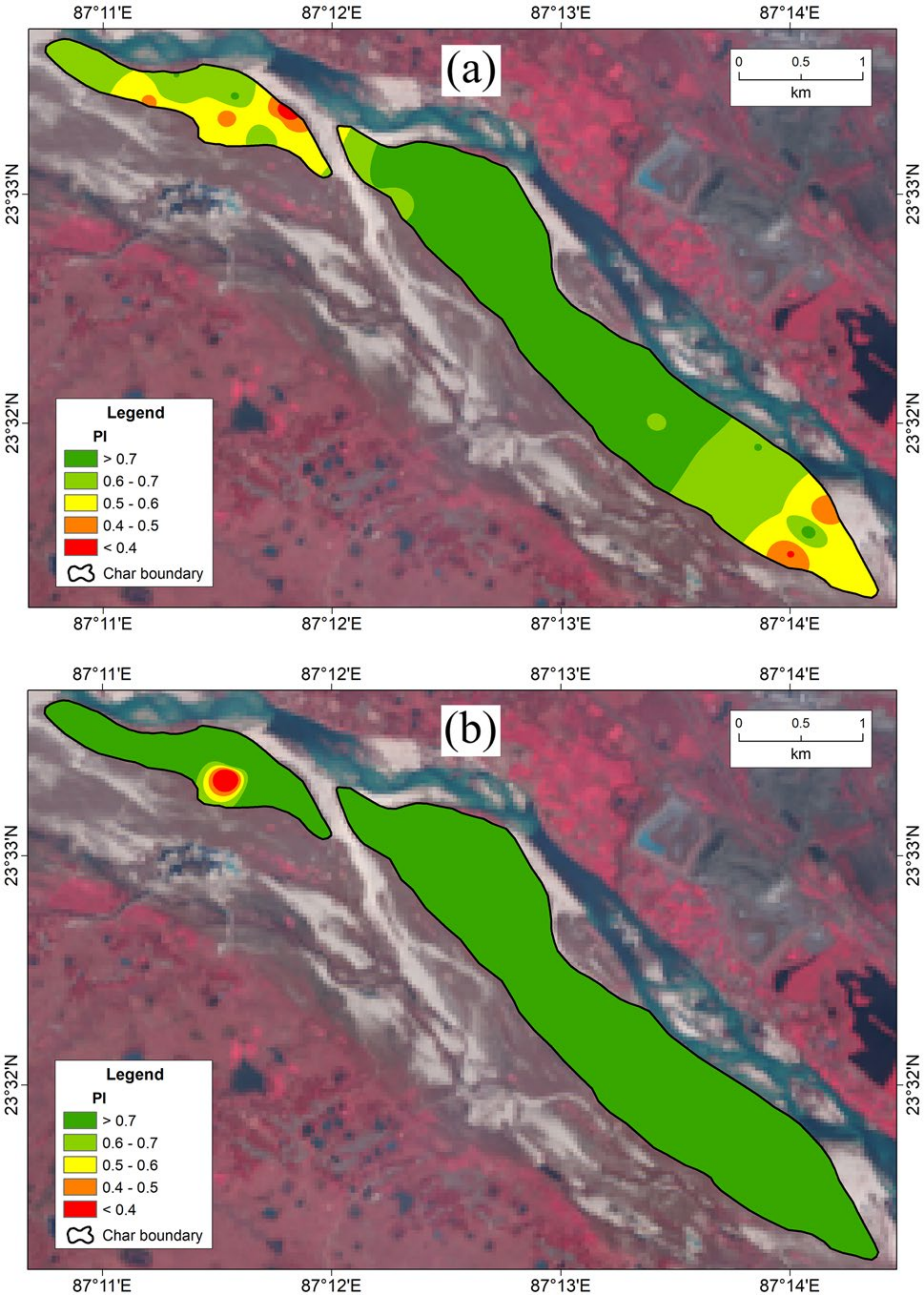
Prioritization of *char* soils based on the concentration of HM (Pi). (**a)** surface soils, (**b)** sub-surface soils (*Source:* prepared by the authors using ArcGIS software-version 10.2).

Prioritization of surface and sub-surface soil is vital for the management and mitigation of soil pollution and its ecological risk using TOPSIS^14^. Pollution and ecological risk assessing indices such as Cd, Cp, EF, I_geo_, MPI, PLI, Er, RI and MRI have been analyzed using the TOPSIS MCDM technique for evaluating the priority of surface and sub-surface soil pollution. The performance rank (Pi) portrays that a higher value of Pi indicates lower soil pollution and vice versa. Hence, rank 1 is the best location while rank 30 is the worst location in terms of soil pollution. The calculated Pi values of surface and sub-surface soils are mentioned in supplementary (Table S4). The final relative closeness score of the ideal solution was found to be 0.29–0.81 with a 0.65 mean value in the surface soils while it was found to be 0.04–0.99 with a 0.94 mean value in the sub-surface soils. These results indicate that the sub-surface soils have lower soil pollution than the surface soils. Furthermore, the final score or Pi values are classified into five groups such as very low (< 0.4), low (0.4–0.5), medium (0.5–0.6), high (0.6–0.7), and very high (> 0.7) (Fig. 10a,b).

### Geostatistical modeling

This study found that all the semi-variogram models (spherical, exponential, Gaussian, and circular) have arisen as best fit models for OK and SK interpolation methods for some elements as there was no difference in the ME, MSE, RMSE, ASR, and RMSSE values (Table S5). It was observed that the circular semi-variogram model of the OK technique is the best-fit model compared to the other two models (SK and IDW) for pH of surface soils while all the studied semi-variogram model of the SK technique is the best-fit model compared to other two models (OK and IDW) for pH of sub-surface soils (Figs. S1 and S2). Similarly, for Cu, the Gaussian semi-variogram model of the OK technique is the best-fit model for surface soils while all the studied semi-variogram models of the SK technique are the best-fit model for sub-surface soils. Inversely, in case of OC, CEC, and Zn, all studied the semi-variogram models of SK, SK and OK interpolation techniques appeared the best-fit model for surface soils while Gaussian, exponential and circular semi-variogram models of SK interpolation technique are the best-fit models for sub-surface soils. Regarding Mn and B, all the studied semi-variogram models of the SK interpolation technique are the best fit for both levels of soils. Moreover, in case of sand, clay, and Fe, the Gaussian semi-variogram model of SK and OK interpolation technique was observed as the best-fit model for both levels of soils but for sand and clay, SK was observed for surface soils. And OK for sub-surface soils. However, in case of Fe, inverse results were found. For moisture, the spherical semi-variogram model of the SK interpolation technique is the best fit for both levels of soil. Further, for EC and Silt, the circular and exponential/Gaussian semi-variogram models of the OK interpolation technique are the best-fit model for surface soils while Gaussian of SK and OK is the best-fit model for surface soils respectively. Hence, based on these results, the kriging interpolation technique (OK and SK) with the studied semi-variogram model provides better performance for each variable (Table 3). Furthermore, 61.54% of surface soil sample variables and 76.92% of sub-surface soil sample variables fit with the SK interpolation technique and the rest with the OK interpolation technique. So, the SK technique is expected as the most accurate interpolation model for OC, CEC, sand, clay, moisture, Cu, Mn, and B in the surface soils while it is for pH, EC OC, CEC, moisture, Cu, Zn, Fe, Mn and B for sub-surface soils as compared to the other studied techniques (OK and IDW). The elements like a nugget, range, partial sill, and lag size value of best-fit semivariogram models are extracted by using ArcGIS (version 10.2). Later, sill, nugget/sill, and the effect of nugget/sill are also calculated from obtained values for this study (Table 3).

**Table 3:** Best-fit semi-variogram models of each element.

Parameters	Models		Best fit models		Nugget		Major range (km)		partial sill		Nugget/sill		Lag size		Sill	
	A	B	A	B	A	B	A	B	A	B	A	B	A	B	A	B
pH	OK	SK	Circular	All	0.35	1.18	3731.46	4478.13	0.00	0.00	0.99	1.00	466.43	559.77	0.36	1.18
EC	OK	SK	Circular	Gaussian	0.00	0.81	334.54	4478.13	0.03	0.07	0.00	0.92	41.82	559.77	0.03	0.88
OC	SK	SK	All	Gaussian	1.07	0.13	4478.13	334.54	0.00	0.98	1.00	0.11	559.77	41.82	1.07	1.10
CEC	SK	SK	All	Exponential	1.00	0.00	4478.13	1223.98	0.00	1.00	1.00	0.00	559.77	153.00	1.00	1.00
Sand	SK	OK	Gaussian	Gaussian	0.56	8.92	2021.25	541.37	0.43	40.34	0.57	0.18	252.66	67.67	0.99	49.26
Silt	OK	OK	Exponential/Gaussian	Gaussian	24.41	5.16	775.76	514.62	0.00	41.37	1.00	0.11	96.97	64.33	24.41	46.53
Clay	SK	OK	Gaussian	Gaussian	0.95	0.59	1446.73	593.07	0.05	3.56	0.95	0.14	180.84	74.13	1.01	4.15
Moisture	SK	SK	Spherical	Spherical	0.54	0.21	334.54	334.54	0.22	0.78	0.71	0.21	41.82	41.82	0.77	0.99
Cu	SK	SK	Gaussian	All	0.08	0.93	334.54	4171.48	0.78	0.00	0.09	1.00	41.82	521.43	0.86	0.93
Zn	OK	SK	All	Circular	0.04	0.00	2031.52	453.40	0.00	0.93	1.00	0.00	253.94	56.67	0.04	0.93
Fe	OK	SK	Gaussian	Gaussian	73.22	0.99	4478.13	2104.87	17.03	0.01	0.81	0.99	559.77	263.11	90.26	1.00
Mn	SK	SK	All	All	1.02	0.99	4478.13	2475.37	0.00	0.00	1.00	1.00	559.77	309.42	1.02	0.99
B	SK	SK	All	All	1.02	1.00	4478.13	4478.13	0.00	0.00	1.00	1.00	559.77	559.77	1.02	1.00

## Discussion

**Figure 11:**
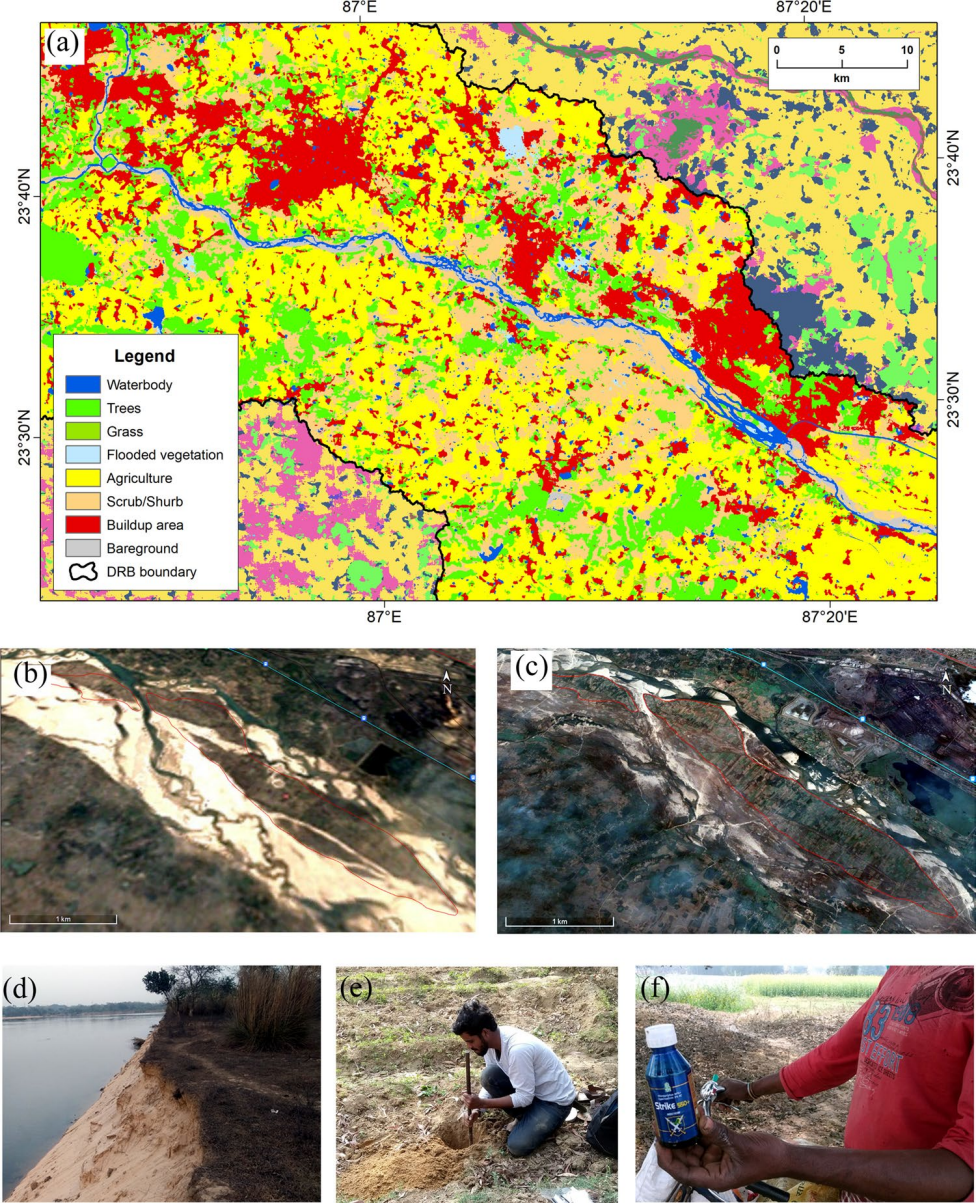
Major land uses and agricultural patterns in the DRB. (**a**) LULC of the *chars* and surroundings during 2020, (**b**) development of *chars* in 1985, (**c**) development of agriculture on *charland* in 2020, (**d**) nature of *char* soils, (**e**) collection of soil samples from *chars*, (**f**) use of pesticides for cultivation (*Source:* (**a**) is prepared by the authors using European Space Agency (ESA) Sentinel-2 imagery- 45Q dated 01 January 2020 (https://www.arcgis.com/apps/instant/media/index.html?appid=fc92d38533d440078f17678ebc20e8e2) and ArcGIS software-version 10.2; (**b**) and (**c**) are prepared from Google Earth imageries dated 31 December (1985 & 2020)-https://earth.google.com/web/; (**d**)–(**f**) are field photographs captured by the authors on 13 February 2021).

The present study assessed the PC parameters of collected surface and sub-surface soils of mid-channel bar’s agricultural fields in the Damodar River for measuring HM pollution and their ecological risks. This study observed that the HM concentration was low as per the permissible limit of HM based on Salem et al.^66^. Moreover, it was also observed that the Zn, Fe, and Mn concentrations in surface soils are relatively low than in the sub-surface soil. The fractionation of the HM may have been influenced by pH, OC, CEC, and moisture which all depend on soil structure^69,70^. This study observed that the surface soils ranged from strongly acidic to slightly acidic and sub-surface soils varied from strongly acidic to neutral. Although, Rani et al.^29^ found the alkaline nature of agricultural soil in Delhi, India and they mentioned that low pH has high solubility of HM in soil. Lower concentrations of pH in soil may decrease HM adsorption and increase soil mobility^70^. Moreover, the study suggests most of the surface soils are contaminated with acidic high metals while most of the sub-surface soils are near natural high metals. Kumar and Singh^10^ found neutrals or alkaline with high metals types of sediment in a tropical Ajay River basin system in India, which is closely associated with the present study findings. The major causes of soil acidity are the excess leaching of nitrate, using nitrogen-based fertilizers in the soil, and removing plant and animal products^71,72^. The leaching process accelerates as a result of the sandy soil. Acidic soil impacts agricultural productivity and sustainable farming system by decreasing the availability of essential nutrients and increasing the toxicity of trace elements^73^. The low concentration of OC in the soils was also observed. In addition, OC and the percentage of soil moisture concentration in surface soils were higher but the CEC was lower than in the sub-surface soils. The CEC in soils indicates that sub-surface soil can hold the exchangeable cations more than surface soil. Yu et al.^70^ mentioned that reducing soil CEC may increase bioavailability in soil. Moreover, due to the sandy soil texture, the concentrations of these physico-chemical parameters in the mid-channel bar soils may be found low as these PC or HM are washed out with run-off as agricultural effluents into the river Damodar (Fig. 11a). These mixing agricultural effluents increase the pollution level of Damodar River water due to the growth of intensive agriculture on the *chars* (Fig. 11b,c) and the threat to the riverine ecology as well as the riverine biota to some extent. Our study findings are supported by Sarkar et al.^52^.

The analysis of HM contamination shows a low contamination level for CF, Cd, and Cp in both the surface and sub-surface soil. Perumal et al.^33^ reported a similar finding in their study on the Thondi coast, Palk Bay, South India. Further, the geoaccumulation index indicates that all the soils from both levels are uncontaminated to moderately contaminated by HM. Our study findings are also supported by Sarma Bora et al.^74^. Although Perumal et al.^33^ found that Fe and Mn were extremely contaminated, Zn was strong to extremely contaminated and Cu was strongly contaminated in their geoaccumulation study. Moreover, Radomirović et al.^75^ found moderately polluted soil by Zn based on their geoaccumulation study at a former painting industry facility. Furthermore, the PI and PLI depict that all the samples (both levels) are unpolluted but surface soil is more polluted than the sub-surface. Hu et al.^11^ also found a safe level of HM pollution in the soil at a coastal industrial city in the Yangtze River Delta, China. The soils have a low level of contamination due to the lower concentration of HM in collected soils. The distribution of HM and their pollution level depend on natural and anthropogenic factors such as rock weathering, urban-industrial wastewater, transportation, and modern agriculture^4,13,33^. Moreover, Wang and Zhang^12^ found that the deposition of HM in urban roadside soil was due to restoring damaged roads and maintaining green belts. However, the present study found that the distribution of HM and their pollution level are influenced by modern agricultural practices, soil structure, precipitation, and river water fluctuations. The HM pollution concentration is lower in the soil profile because of two factors—(1) sandy soil of the *char* and (2) frequent *char* inundation. The sandy soils absorb the pollutants and infiltrating water through the soil layers leaches pollutants down to the deeper soil layer or the river (Fig. 11d,e). A similar finding was reported by Ciszewski et al.^76^. Moreover, the low-lying parts of the *chars* frequently become inundated by monsoon floods almost every year that disturb the geo-accumulation process resulting in a lesser pollution concentration. Our study findings are also supported by Sarkar et al.^52^. HM pollution of the soil though low is contributed mainly by the *charland* agricultural practice pre-monsoon season because of the huge application of chemical fertilizers and pesticides for agricultural production (Fig. 11f).

The study reveals that the soils have a low ecological risk and low potentiality of ecological risk. However, the ecological risk is comparatively higher in the surface soil than in the sub-surface soil due to the lower concentration of HM in the sandy soils. Additionally, the potential ecological risk of HM is ranked as Cu > Zn > Mn for both levels of the soil. Liu et al.^77^ found that most of the HM (As, Cr, Cu, Ni and Zn) had a low potential ecological risk in coal mining areas in China. Additionally, Perumal et al.^33^ also found a low-potential ecological risk from their studied HM and considerable ecological risk from Cu and Zn, and similar results were also found by Sarma Bora et al.^74^. Besides, Radomirović et al.^75^ found moderate pollution and ecological risk for most soils at a former painting industry facility. Moreover, as per the enrichment factor, the HM is ranked as Mn > Zn > Cu for both levels of soil. The HM enrichment in sub-surface soil ranges from very severe to extremely severe while they range from severe to extremely severe for the surface soils. Perumal et al.^33^ found no enrichment for Mn, Fe, and Cu but Radomirović et al.^75^ found a moderate soil enrichment for Zn in the soil. Sarma Bora et al.^74^ found significant enrichment for Cu and Zn in solid waste dumping site soil near the Morabharal River, Tezpur town, India.

Moreover, the final relative closeness score of the ideal solution indicates that the sub-surface soils have lower soil pollution than the surface soils in the present investigation. Further from the geostatistical modeling, it was observed that the SK technique was expected to be the most accurate interpolation. Islam et al.^18^ found that the SK technique was the most accurate interpolation model for As, Mn, Zn, and AI while the OK technique for Fe and IDW with power1 for Ba. The kriging models (SK and OK) found as the most accurate interpolation technique for the spatial distribution map of all elements. Islam et al.^18^ also found a similar result for geostatistical modeling analysis in their study in the Rangpur district, Bangladesh. Moreover, the Gaussian semivariogram model was observed as the best-fit semivariogram model among the other semivariogram models (circular, spherical and exponential) studied.

Hence, this investigation would be helpful for decision-making for sustainable agricultural development, improving the degrading riverine ecology and better representation and map-making processes development. Although this present study has some limitations. The main limitation of the study is data unavailability of other trace elements or HM like arsenic (As), nickel (Ni), cadmium (Cd), molybdenum (Mo), chromium (Cr), mercury (Hg), lead (Pb), fluorine (F), and selenium (Se) and lack of sufficient fund for testing these trace elements. Despite those drawbacks, the finding of this study may be helpful to stakeholders including regional planners, and agricultural engineers in the DRB.

## Conclusions

The results of this study portray the nature of PC parameters, HM pollution, and ecological risk in mid-channel bar surface and sub-surface soils from the DRB. This study also shows the best-fit spatial distribution models for the studied variables from both levels of soil. The major findings are mentioned below.

- The concentration of mean pH shows that surface soils are moderately acidic and the sub-surface soils are slightly acidic. Moreover, the Ficklin diagram shows that 76.67% of surface soil is acid-high metal while 83.33% of sub-surface soils are near natural high metal.
- Shepard Triangle Diagram shows that 83.34% of surface soils and 90% of sub-surface soils are sandy loam in the mid-channel bar of Damodar River.
- Similar results were observed as the HM concentration in soils was low, the contamination level was also low (based on the CF, Cd, and Cp values) and the PI and PLI also indicate that all the samples (both levels) were the unpolluted or low level of pollution but the geoaccumulation index shows that all the soils are uncontaminated to moderately contaminate;
- The potential ecological risk index also shows that all the soils (both levels) have a low level of ecological risk. Inverse results were observed between potential ecological risk and enrichment as the potential ecological risk of HM is ranked as Cu > Zn > Mn while enrichment as Mn > Zn > Cu for both levels of soils.
- Moreover, TOPSIS shows the sub-surface soils have lower soil pollution than the surface soils of the studied *chars*.
- Geostatistical modeling analysis shows that the SK technique was expected as the most accurate interpolation model as 61.54% and 76.92% of the surface and sub-surface soil sample’s variables fit with the SK interpolation technique compared to other interpolation techniques (OK and IDW) and Gaussian semi-variogram model was the best-fit semi-variogram model among the other semivariogram models (circular, spherical and exponential) studied.
- The results of numerous indices show that the pollution or contamination level and ecological risk from surface soil are more as compared to the sub-surface soil while the enrichment, geoacumulation, and potential contamination of sub-surface soils HM are high than the surface. Moreover, the sub-surface soil is more sandy than the surface soil and the concentration of Cu and pH is high than the surface soil. The SK technique and Gaussian semi-variogram model was expected to better fit the model as the most accurate interpolation model for sub-surface soil variables compared to the surface soil variables.

## Supplementary Information

Supplementary Information.

## Data Availability

The datasets used and/or analyzed during the current study are available from the corresponding author (AI) on reasonable request.
