# Supplementary material for: Assessment of soil heavy metal pollution and associated ecological risk of agriculture dominated mid-channel bars in a subtropical river basin
Source: Sci Rep. 2023 Jul 9;13:11104. doi: 10.1038/s41598-023-38058-0 (PMC10330174; doi:10.1038/s41598-023-38058-0)
Supplement: Supplementary file 1 — Supplementary Information. [file 41598_2023_38058_MOESM1_ESM.docx]

**Supplementary materials**

**Supplementary Text**

**ST 1. Principles and algorithms of geostatistical models and selection of the best-fit model**

The geostatistical model used in the present study is related to the semi-variogram and associated measurements. The structural features of a semi-variogram model are used to assess the value of distinct variables at a non-sampled location based on the data set of distinct variables^1^. Firstly, the semi-variogram algorithm ᵞ(h) is computed using Eq. 1^1^.

ᵞ(h) =$\frac{1}{2n}\sum_{i=1}^{n} \left[ z\left( x_{i} \right)-z(x_{i}+h) \right]^{2}$ (1)

Where n = pair numbers of sample points divided by the standard distance called lag h z(x_i_) = z variable value at x_i_ location point.

Semi-variogram ᵞ(h) is defined through the nugget (C0), the sill (C), and the range (A0) elements and tested as the best-fit model. Secondly, the best-fit semi-variogram model like circular, exponential, spherical, and Gaussian is selected based on the root mean square error (RMSE) value. The semi-variogram models (circular, exponential, spherical, and Gaussian) have been used to predict the spatial autocorrelation of variables. The nugget denotes the random variation level in the data set and the sill is the variance of the random variable. Kriging models are classified into several models such as simple, ordinary, indicator, and universal, probability, disjunctive, areal interpolation empirical, and Bayesian kriging. However, in the present investigation, OK and SK with IDW have been comparatively studied for representing the spatial variation of the variables (pedo-chemical parameters). The OK model is calculated using Eq. 2.

ẑ (x_o_) = $\sum_{i=1}^{n} \lambda_{i}z(x_{i})$ (2)

Where ẑ stands for the measured value at the sampled point x_o_, z for the observed value at point x_i,_ λ_i_ for the weight assigned to the point, and n for the sampled number used for the prediction. The SK model is derived using Eq. 3.

ẑ (x_o_) = $m+\sum_{i=1}^{n} \lambda_{i}[z\left( x_{i} \right)-m]$ (3)

Where m stands for the mean and all other variables are mentioned in Eq. 2.

In the IDW method, the weight (λ_i_) depends on the distance to the estimation location. The distance between the sample location and the estimation point is created based on the weight. The power of weight controls the weight, for example, if the power is lesser then the point’s effect on the distance is lesser than expected. In the IDW model, the weight value increases with decreasing distance from the prediction point and the total weights (λ_i_) are equal to 1. The IDW model is formulated by Eq. 4.

λ_i_ = $d_{i0}^{-p}/\sum_{i=1}^{n} d_{i0}^{-p}$ (4)

Where d_i0_ denotes the distance between the sample location and prediction points. The distance becomes smaller when the weight is increased exponentially by a power parameter of p. We used power parameters (p) 1 and 2 for comparing the effect of different power parameters. Among the three models, IDW is used to create a spatial distribution map from the variables (PC parameters) because of better representation for this study.

Thirdly, a cross-validation technique is used for comparing the numerous interpolation techniques and finding an optimal model for each variable. For determining the best-fit model, the semivariogram techniques are tested for each selected variable. For providing the best prediction of spatial distribution, the selected interpolation model is applied for each data set. After that, mean square error (MSE), mean error (ME), root mean square error (RMSE), root mean square standardized error (RMSSE), and average standard error (ASR) are evaluated to find out the best-fit model. RMSE is used to determine the best-fit model with the most appropriate prediction results. These errors are assessed using Eq. 5 to 9.

ME = $\frac{1}{n}\sum_{i=1}^{n} (p_{i}- o_{i})$ (5)

RMSE = $\sqrt{\left[ \frac{1}{n}\sum_{i=1}^{n} {(p_{i}- o_{i})}^{2} \right]}$ (6)

MSE = $\frac{1}{n}\sum_{i=1}^{n} ({ps}_{i}- {os}_{i})$ (7)

RMSSE = $\sqrt{\left[ \frac{1}{n}\sum_{i=1}^{n} {({ps}_{i}- {os}_{i})}^{2} \right]}$ (8)

ASE = $\sqrt{\left[ \frac{1}{n}\sum_{i=1}^{n} ({p_{i}}^{-^{(\sum_{i=1}^{n} p_{i})}}/{n)}^{2} \right]}$ (9)

Where n stands for the number of the observed point, o and p for the observed and predicted values at i^th^ location, os for the observed standardized score, ps for the predicted standardized score. When the cross-validation is over, the geostatistical models are portrayed with the graphical representation of the distribution of soil sample variables (PC parameters). According to Islam et al.^1^, a robust and accurate model is selected when the value of ME and MSE is close to zero, RMSE and ASR are minimum and RMSSE is close to 1.

**Reference(s)**

1. Islam, T. A. R. M., Shen, S., Bodrud-Doza, M., Atiqur Rahman, M. & Das, S. Assessment of trace elements of groundwater and their spatial distribution in Rangpur district, Bangladesh. *Arab. J. Geosci.* **10**(4), 1-14. <https://doi.org/10.1007/s12517-017-2886-3> (2017).

**Supplementary Figures**

**pH**: best fit model for pH (OK with Circular)


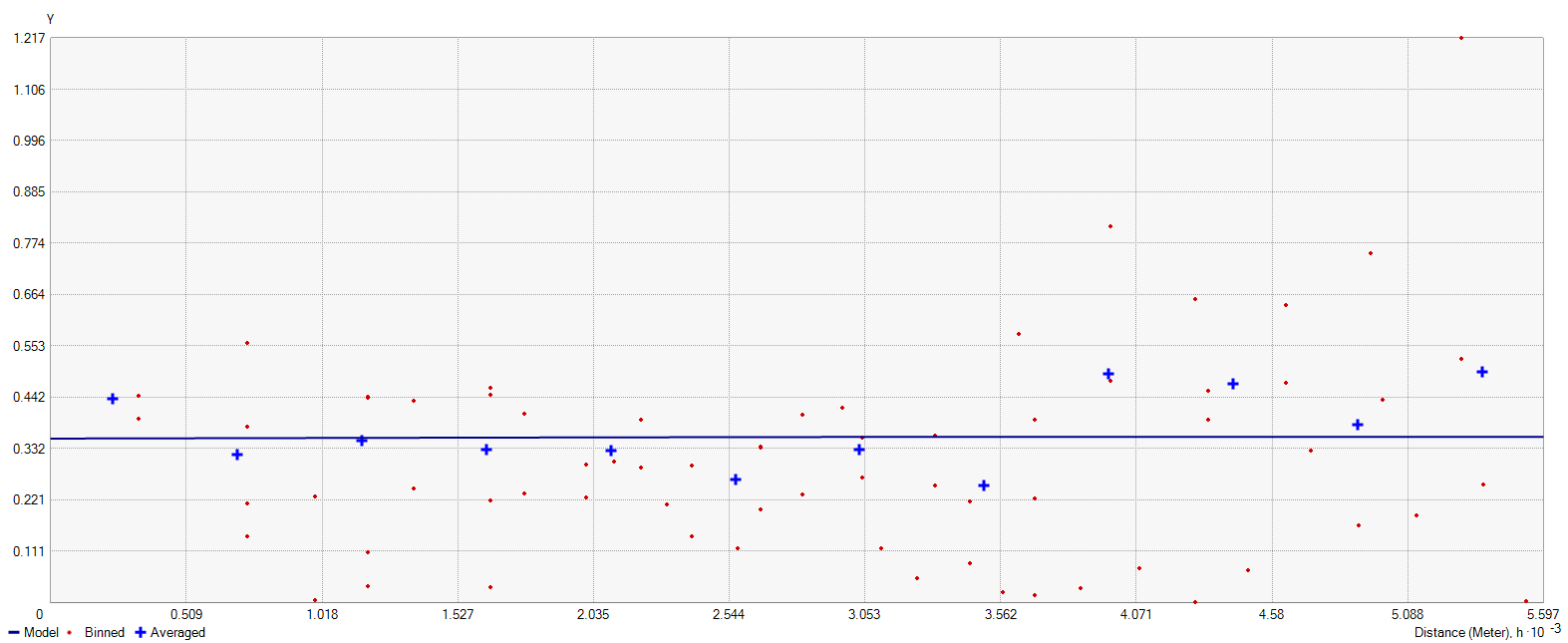


**EC**: best fit model for EC (OK with circular)


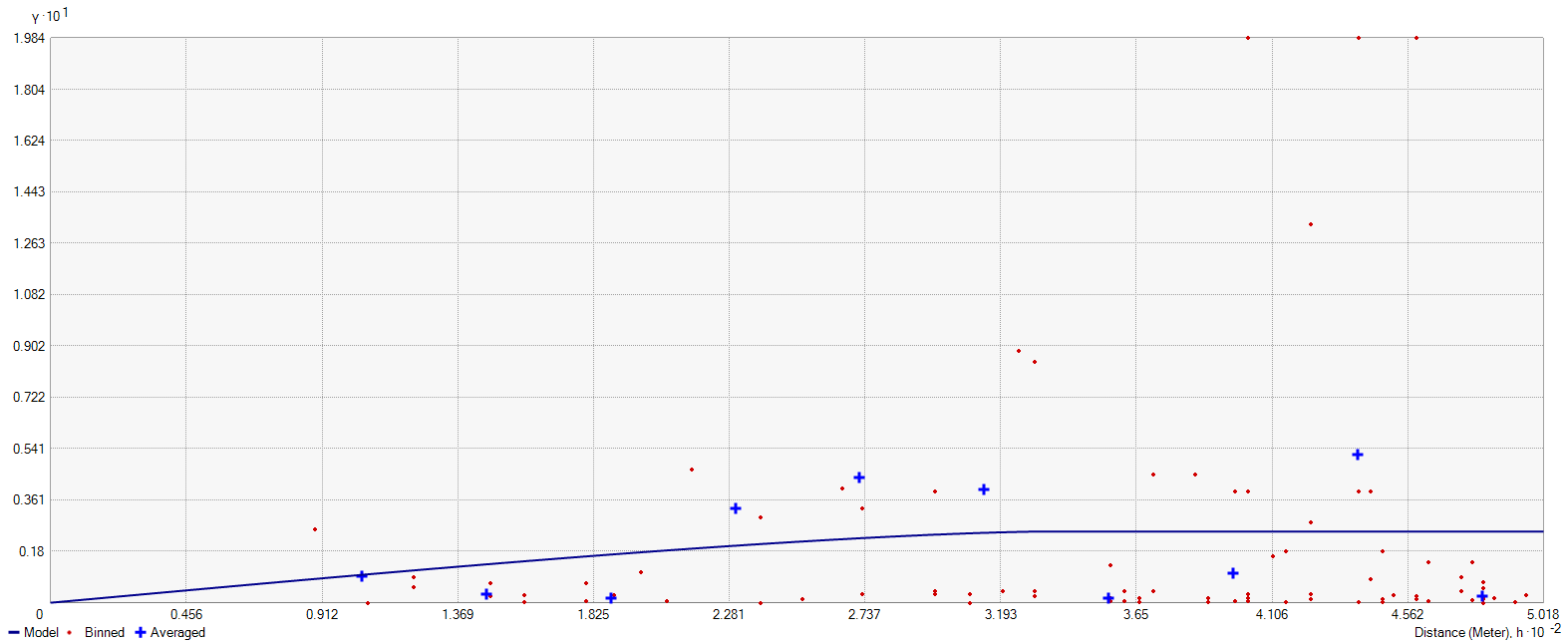


**OC**: best fit model for OC (SK with all)


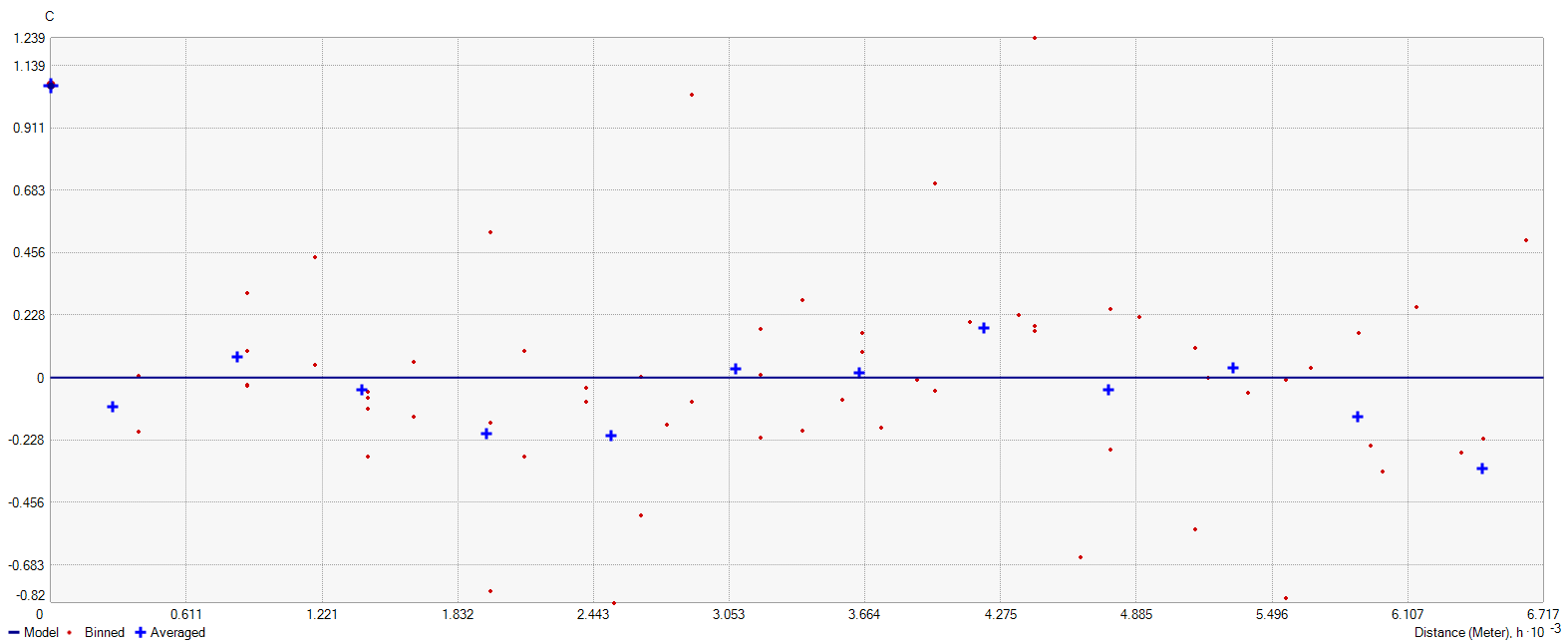


**CEC**: best fit model for CEC (SK with all)


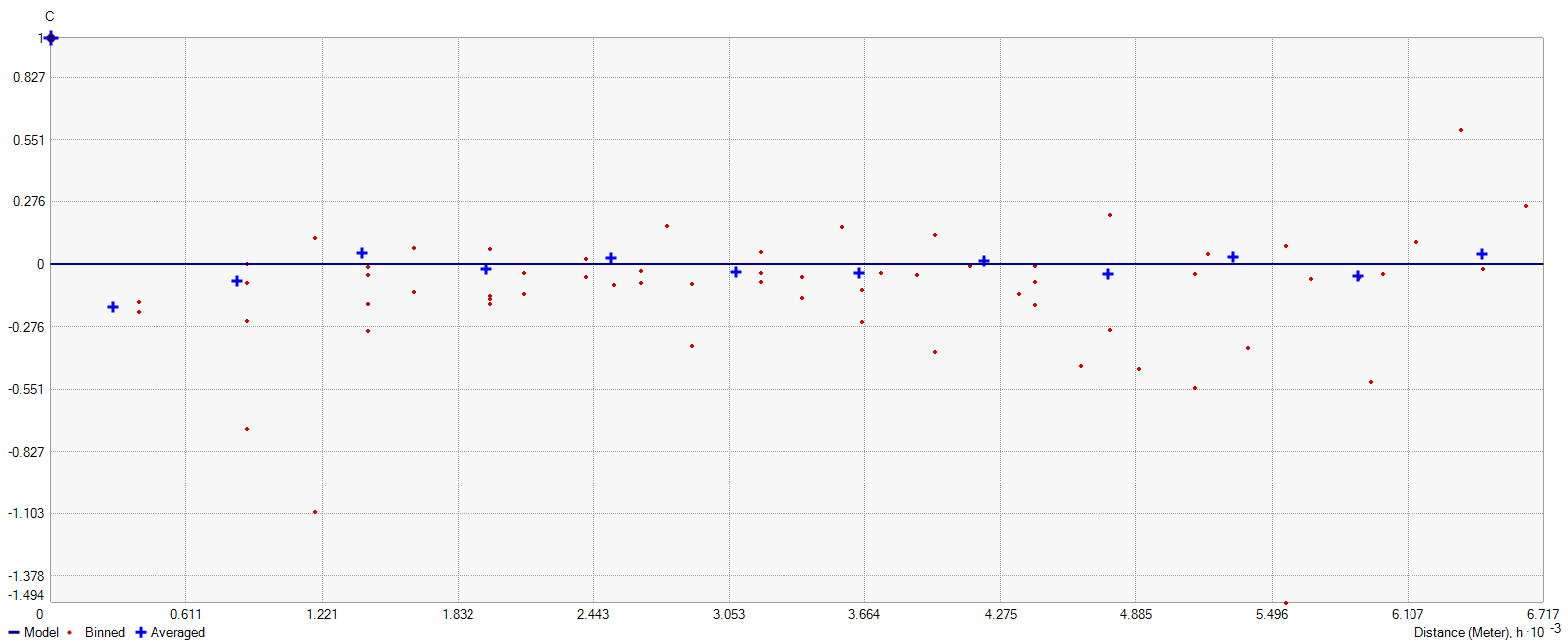


**Sand**: best fit model for Sand (SK with Gaussian)


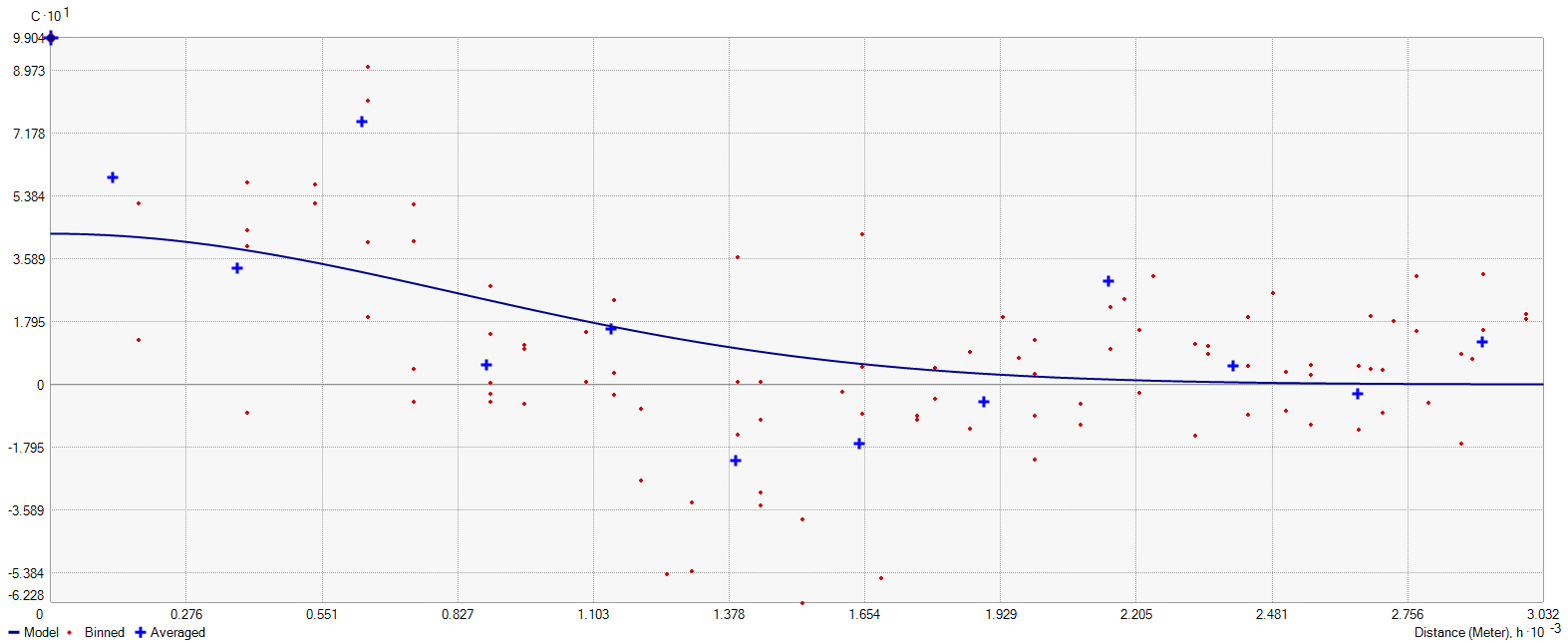


**Silt**: best fit model for Silt (OK with exponential/Gaussian)
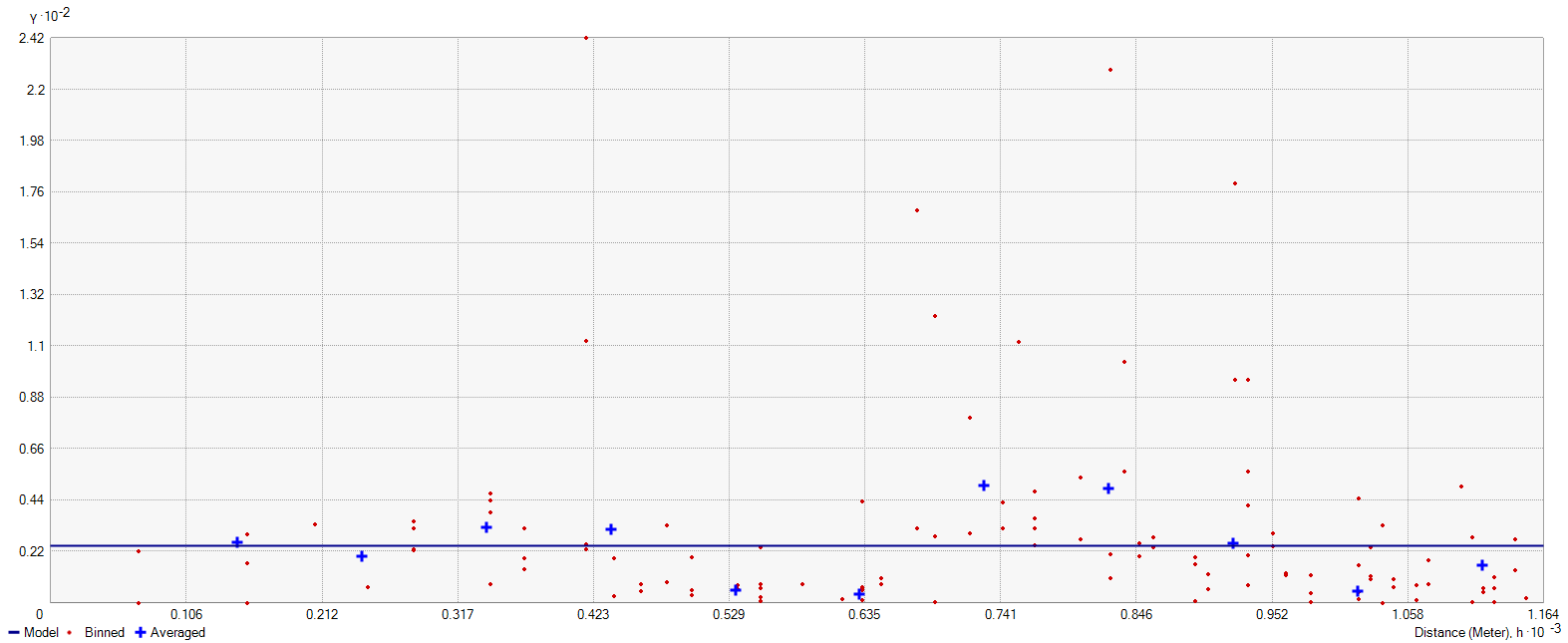


**Clay**: best fit model for Clay (SK with Gaussian)


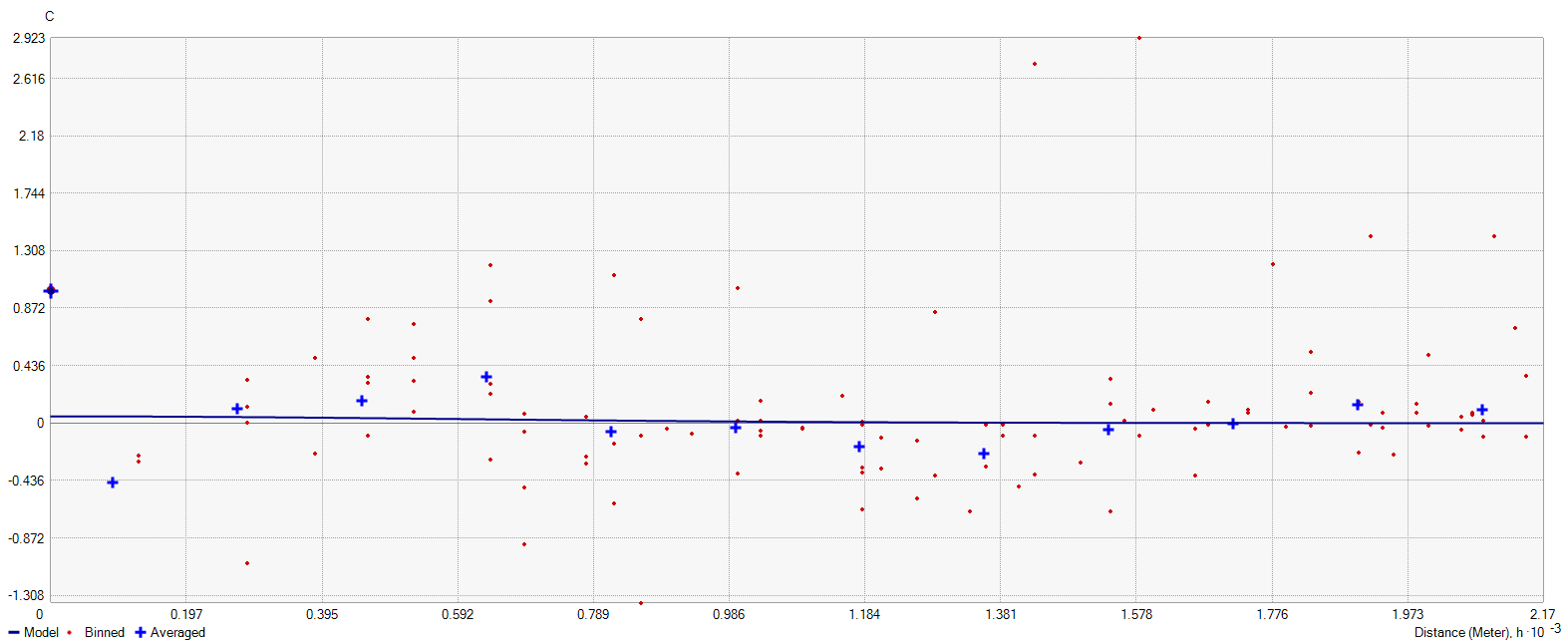


**Moisture**: best fit model for Moisture (SK with spherical)


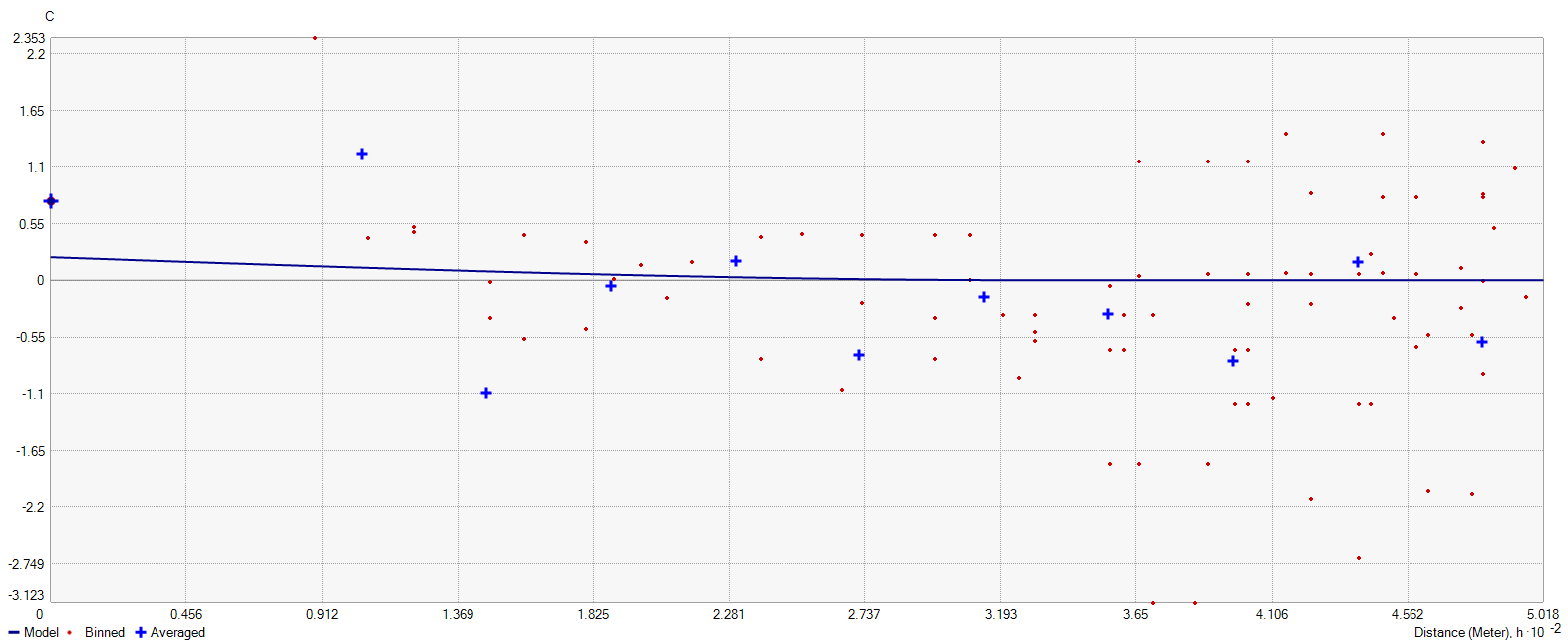


**Cu**: best fit model for Cu (SK with Gaussian)


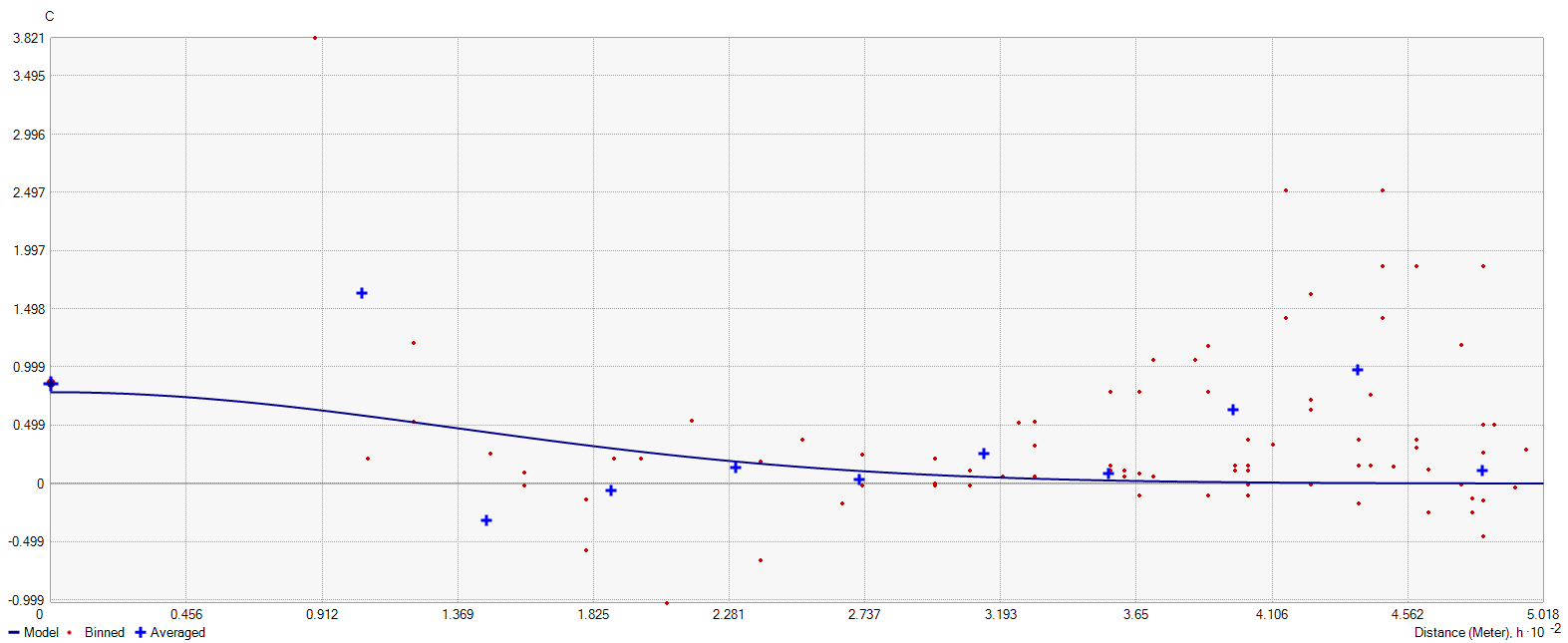


**Zn**: best fit model for Zn (OK with all)


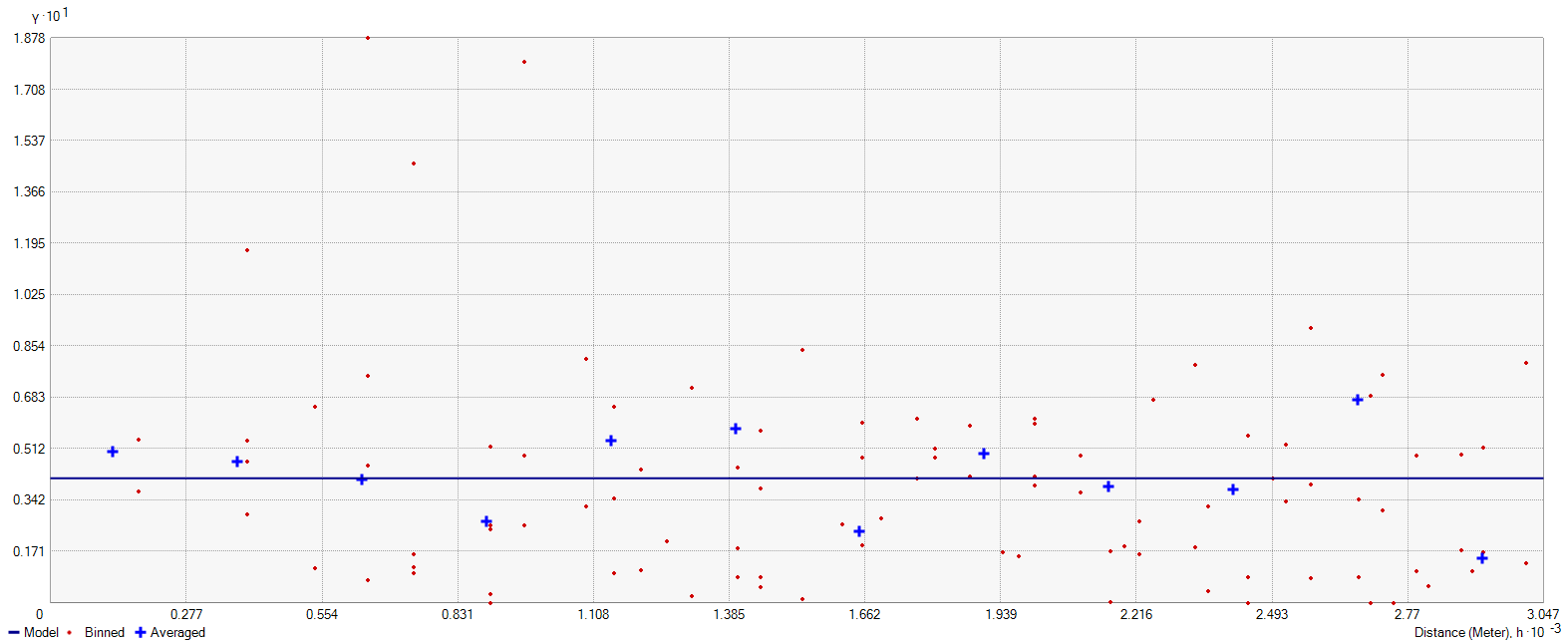


**Fe**: best fit model for Fe (OK with Gaussian)


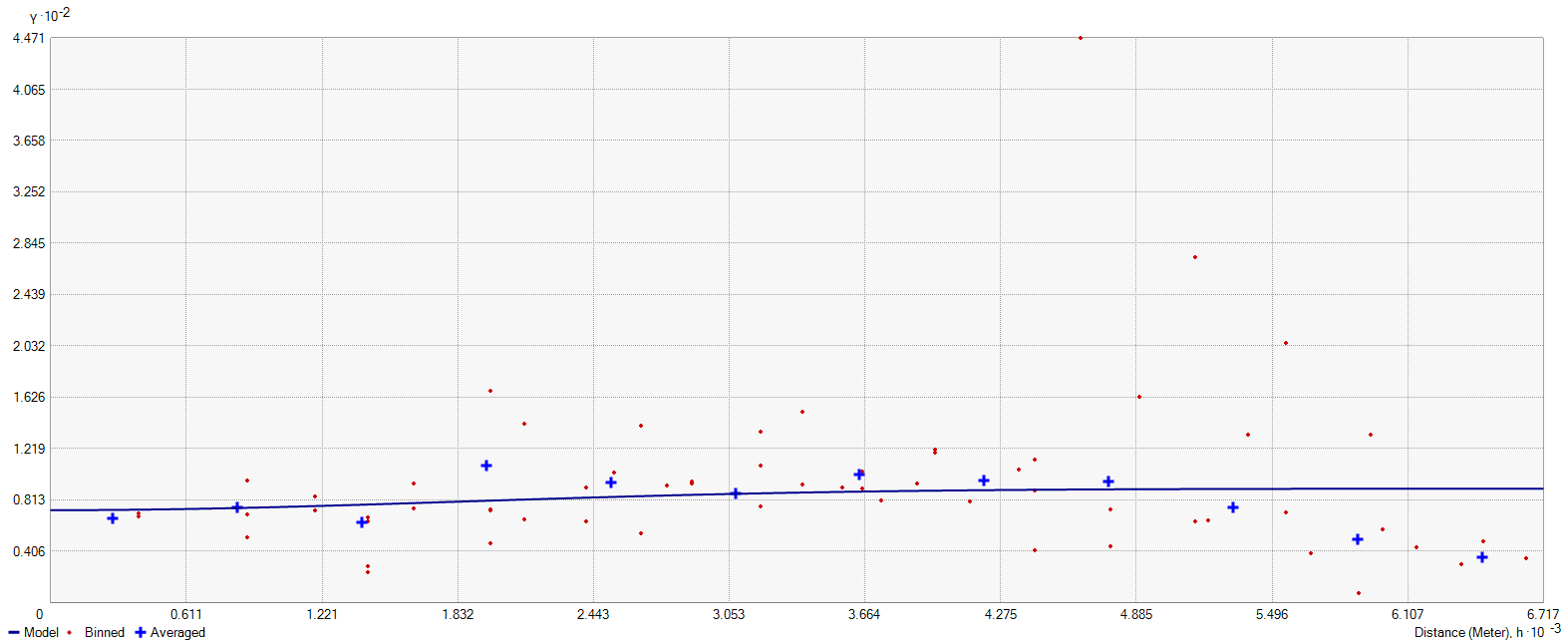


**Mn**: best fit model for Mn (SK with all)


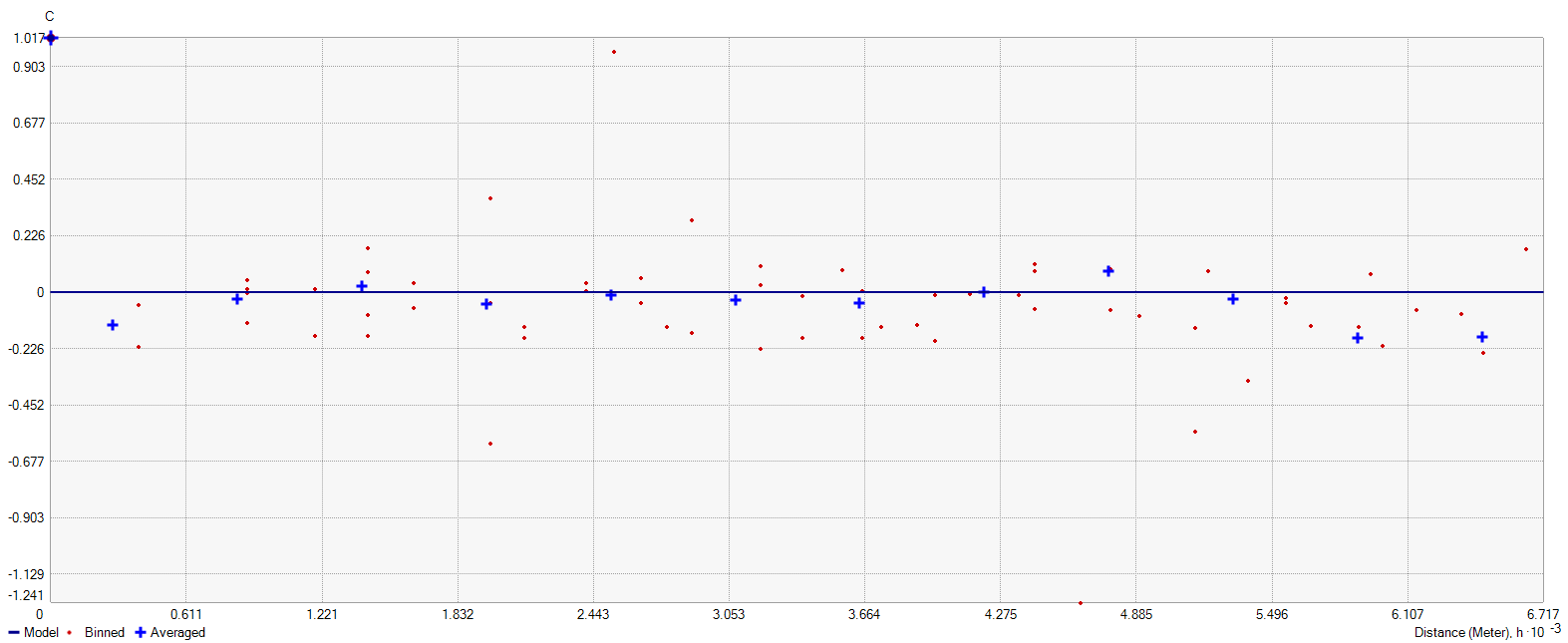


**B**: best fit model for B (SK with all)


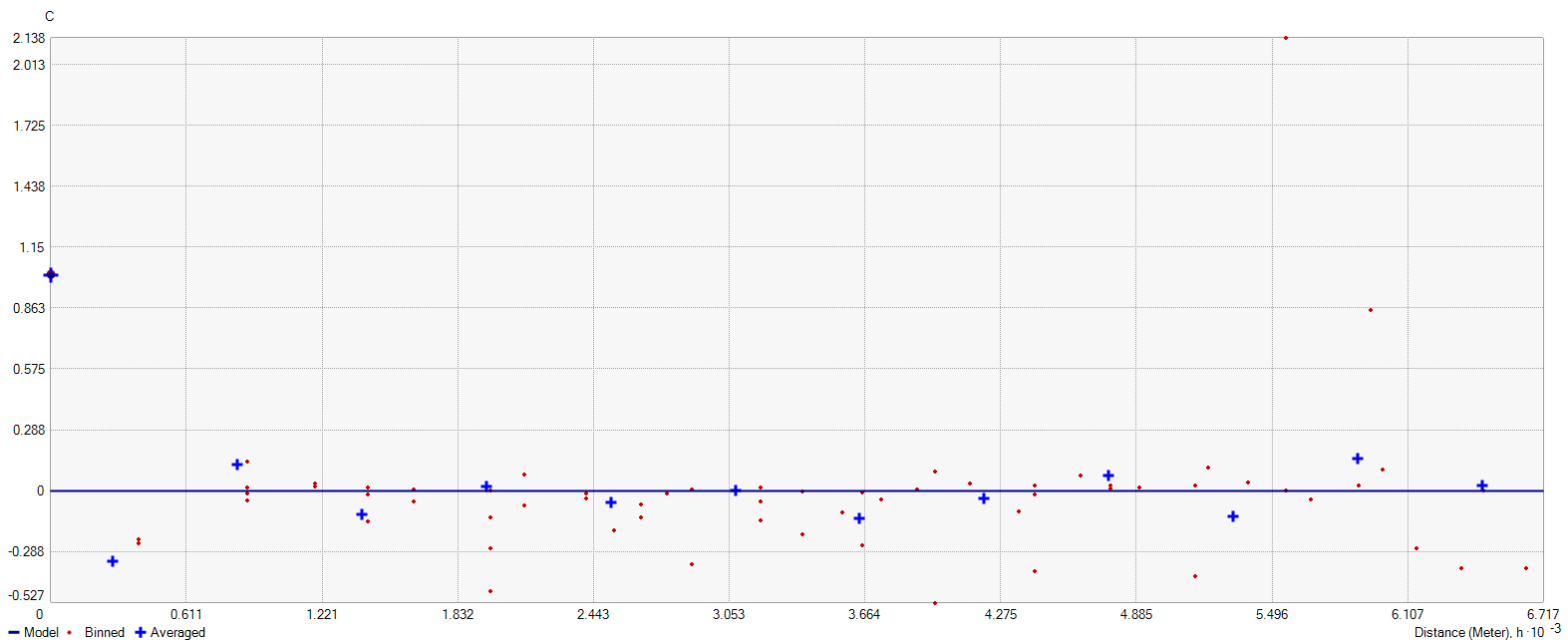


**Figure S1** Semi-variogram model fitting for various pedo-chemical parameters for surface soils

**pH**: best fit model for pH (SK with all)


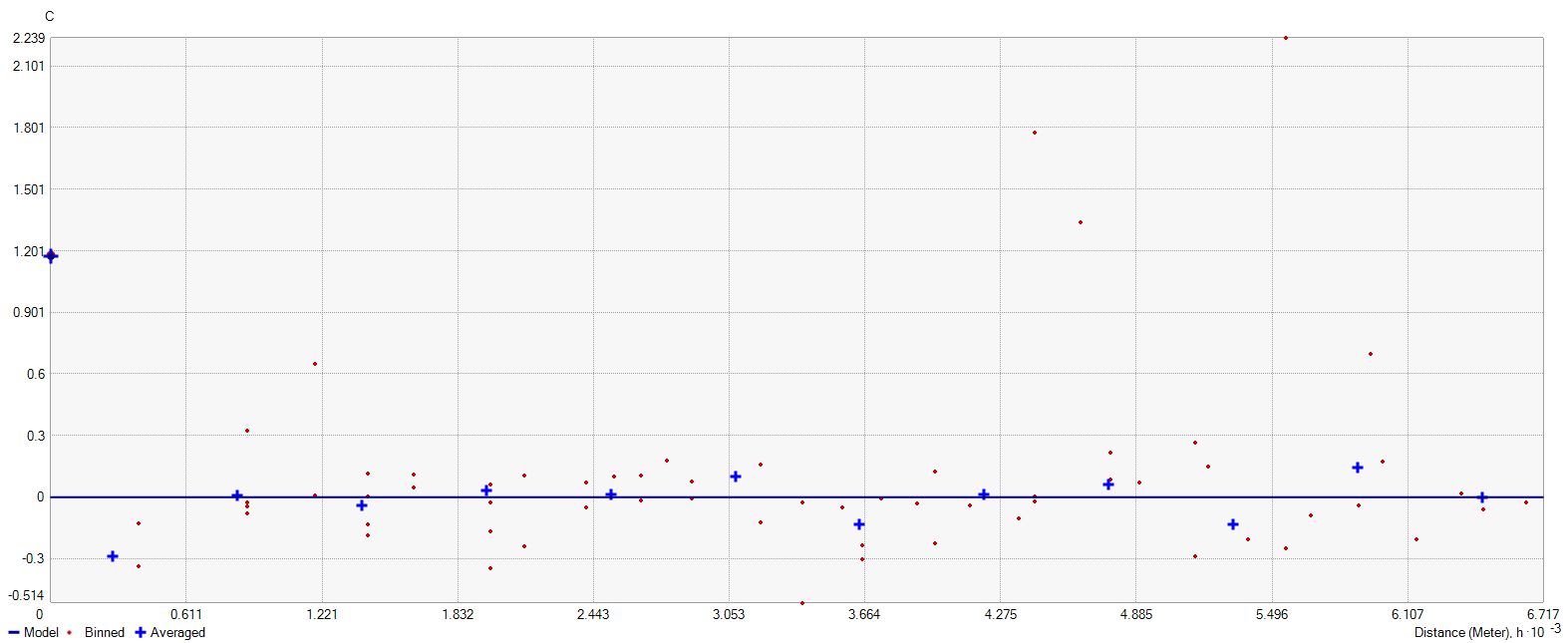


**EC**: best fit model for EC (SK with Gaussian)


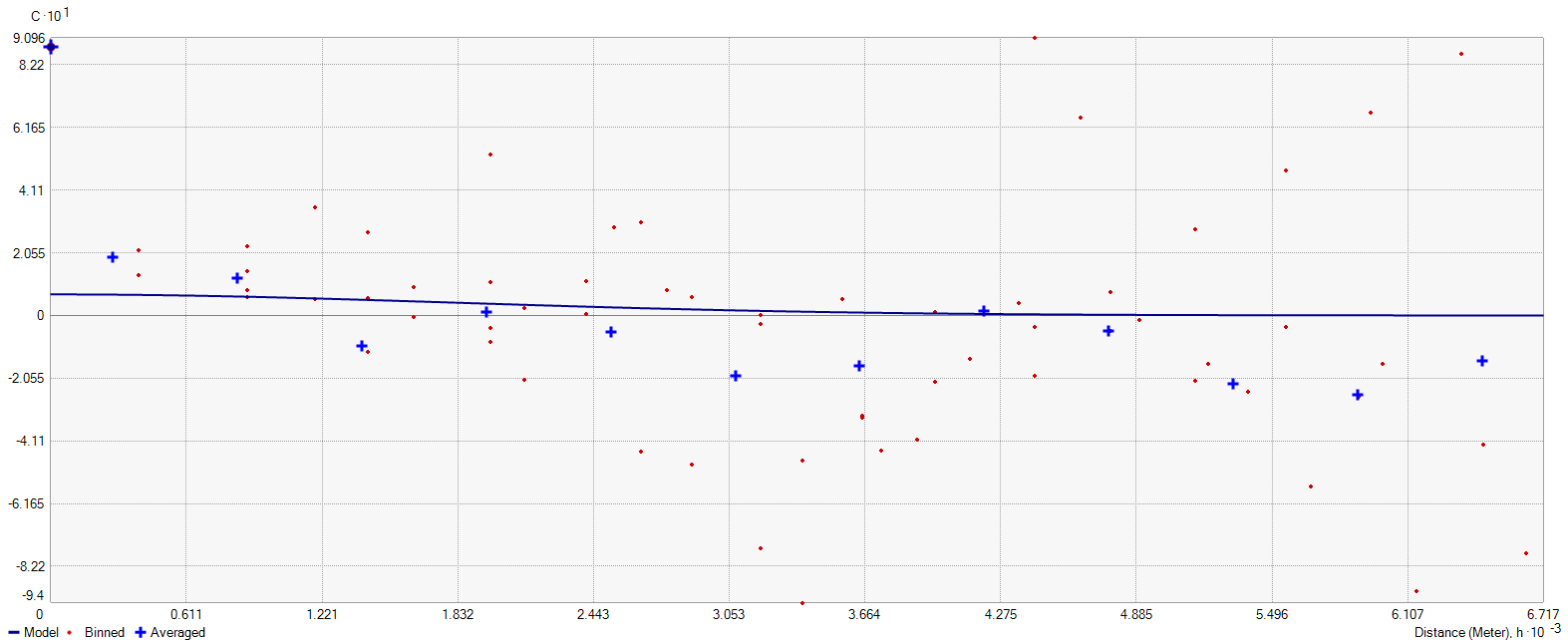


**OC**: best fit model for OC (SK with Gaussian)


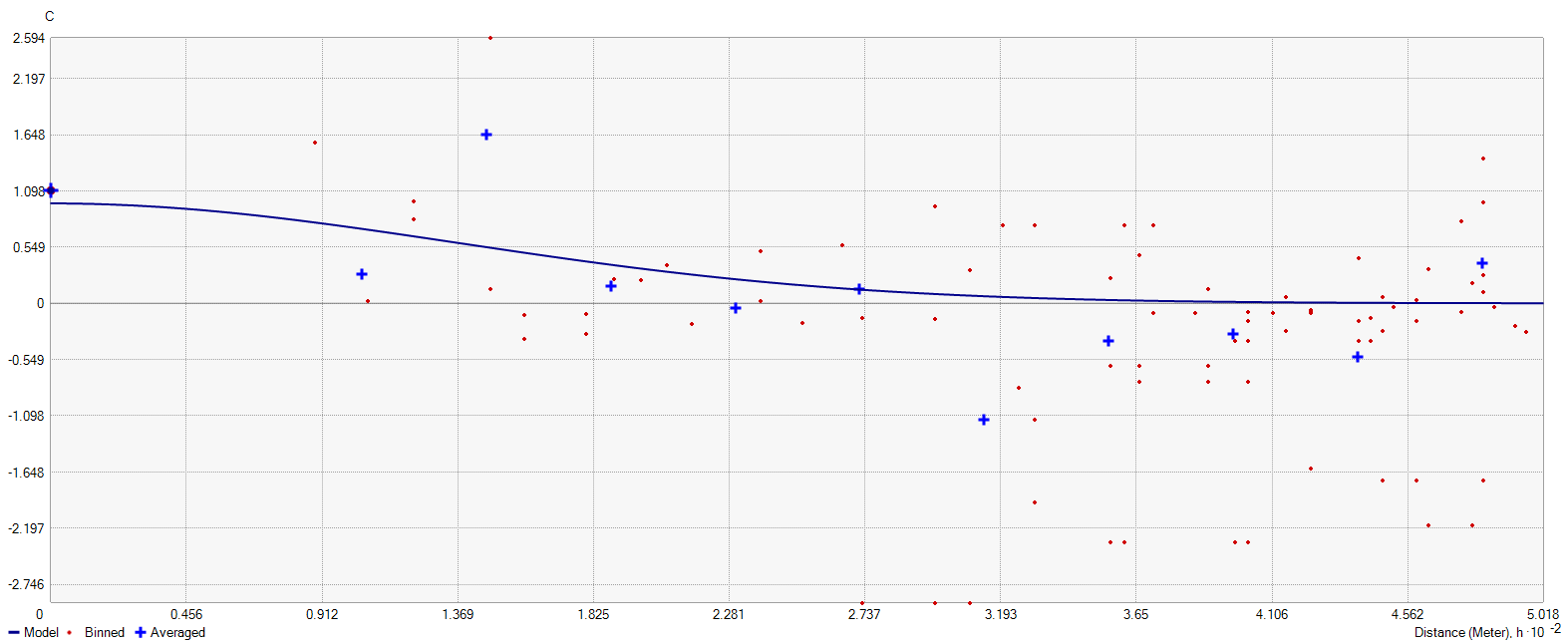


**CEC**: best fit model for CEC (SK with exponential)


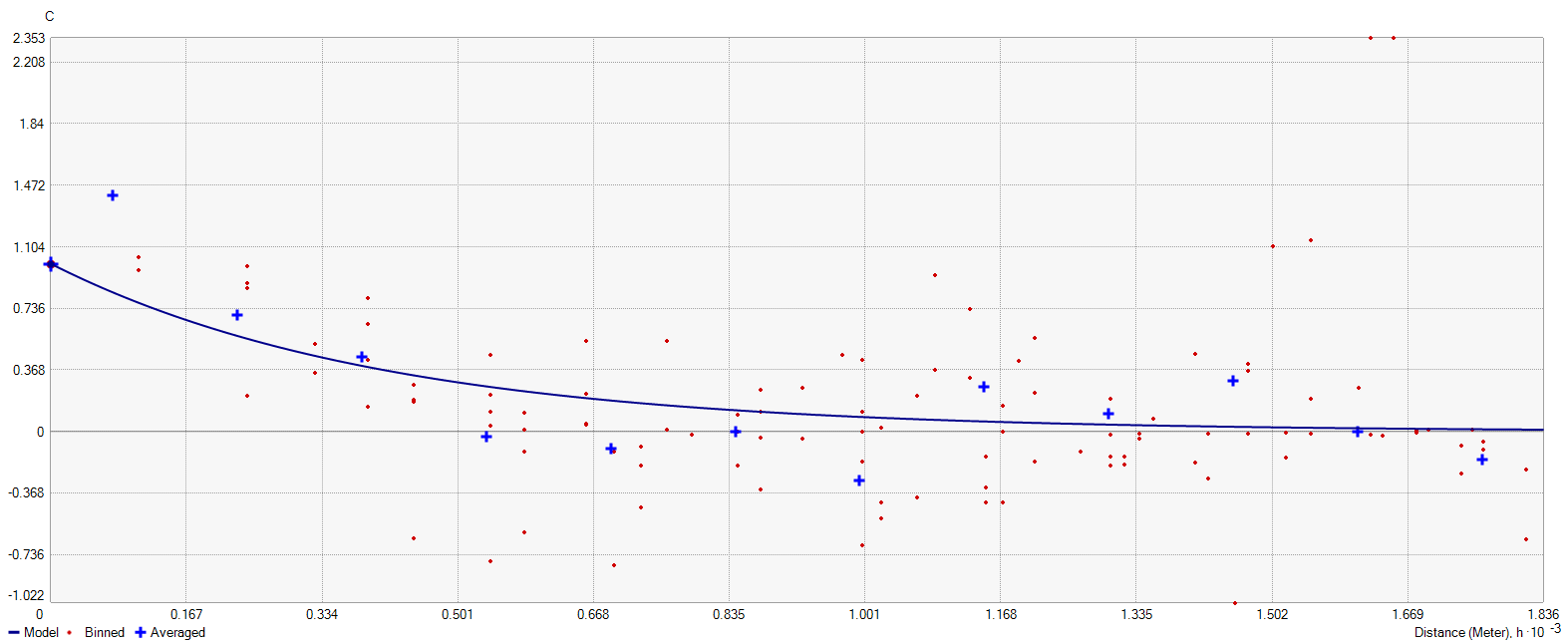


**Sand**: best fit model for Sand (OK with Gaussian)


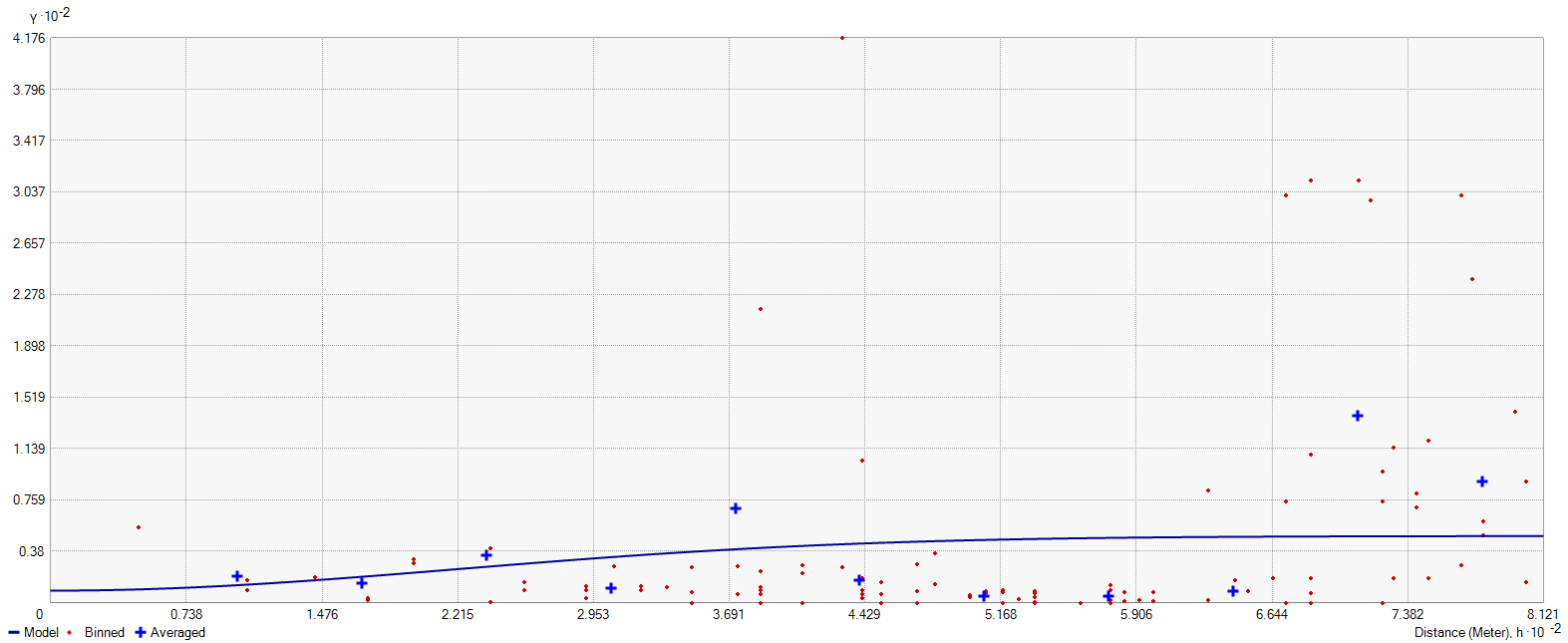


**Silt**: best fit model for Silt (OK with Gaussian)


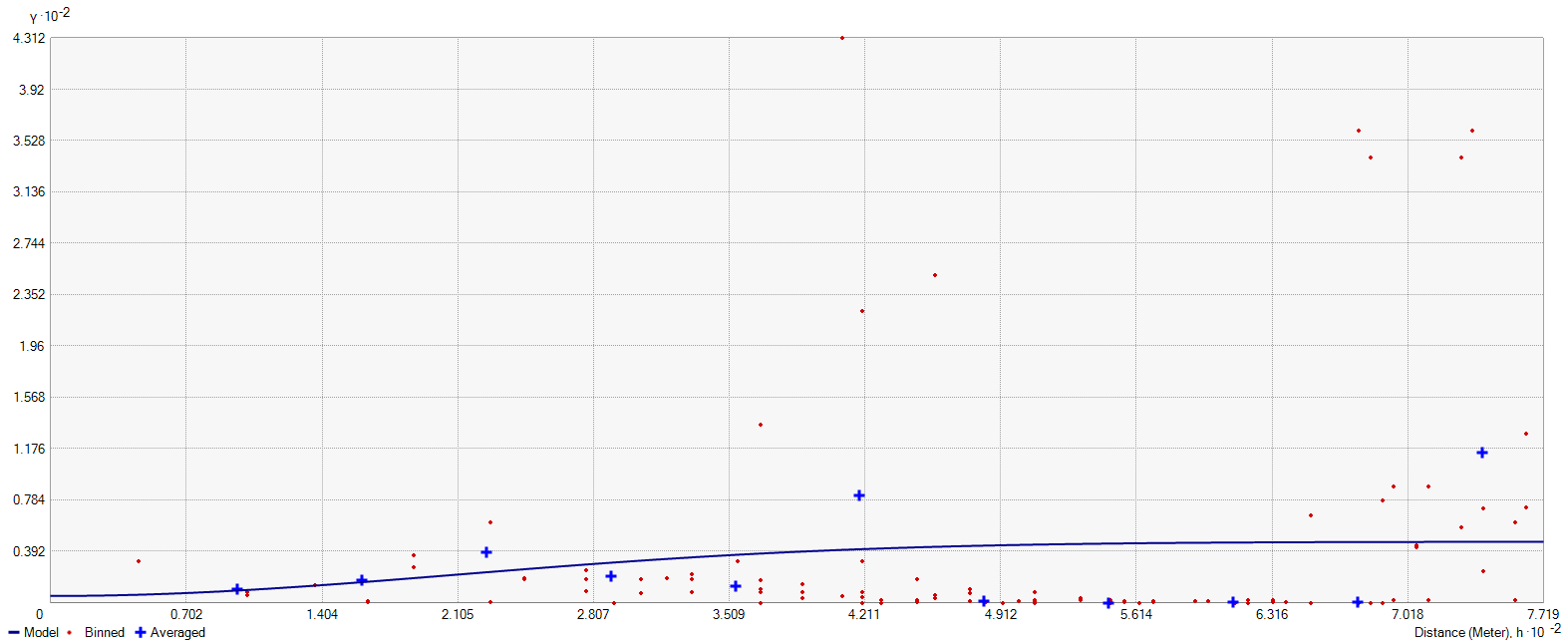


**Clay**: best fit model for Clay (OK with Gaussian)


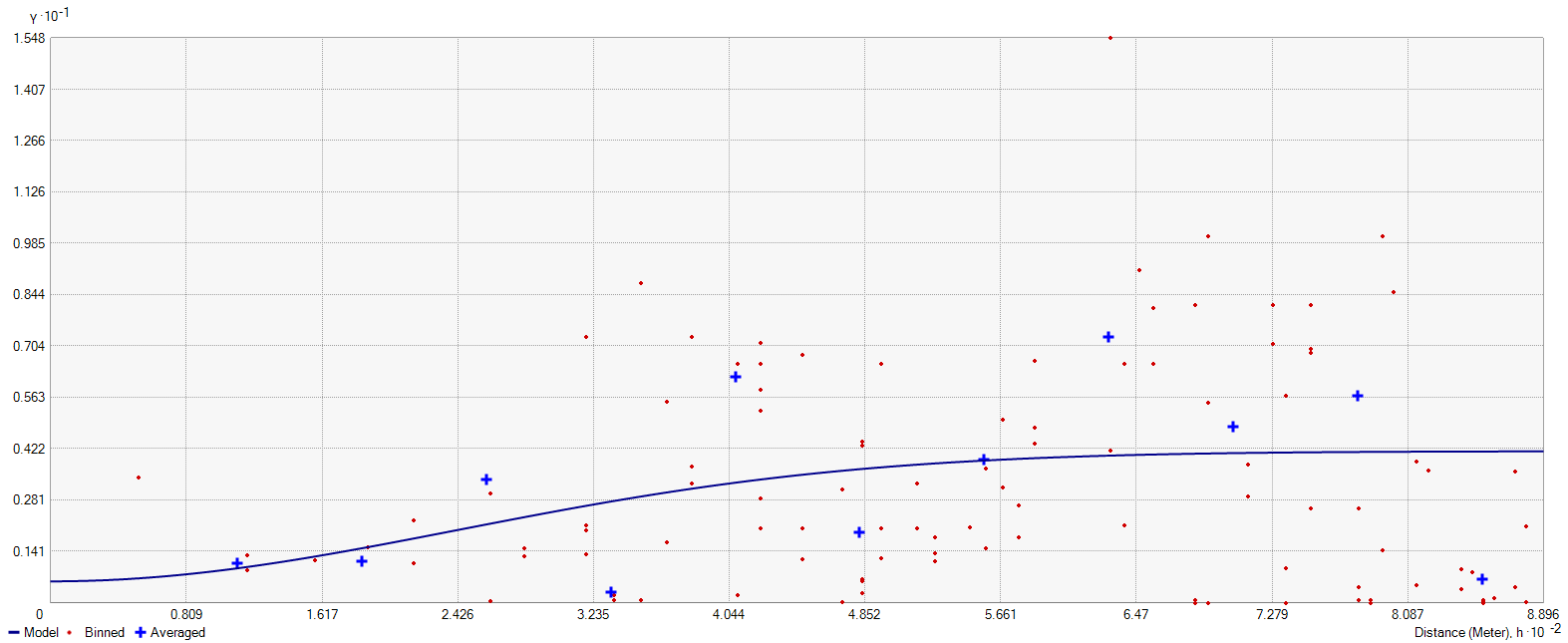


**Moisture**: best fit model for Moisture (SK with spherical)


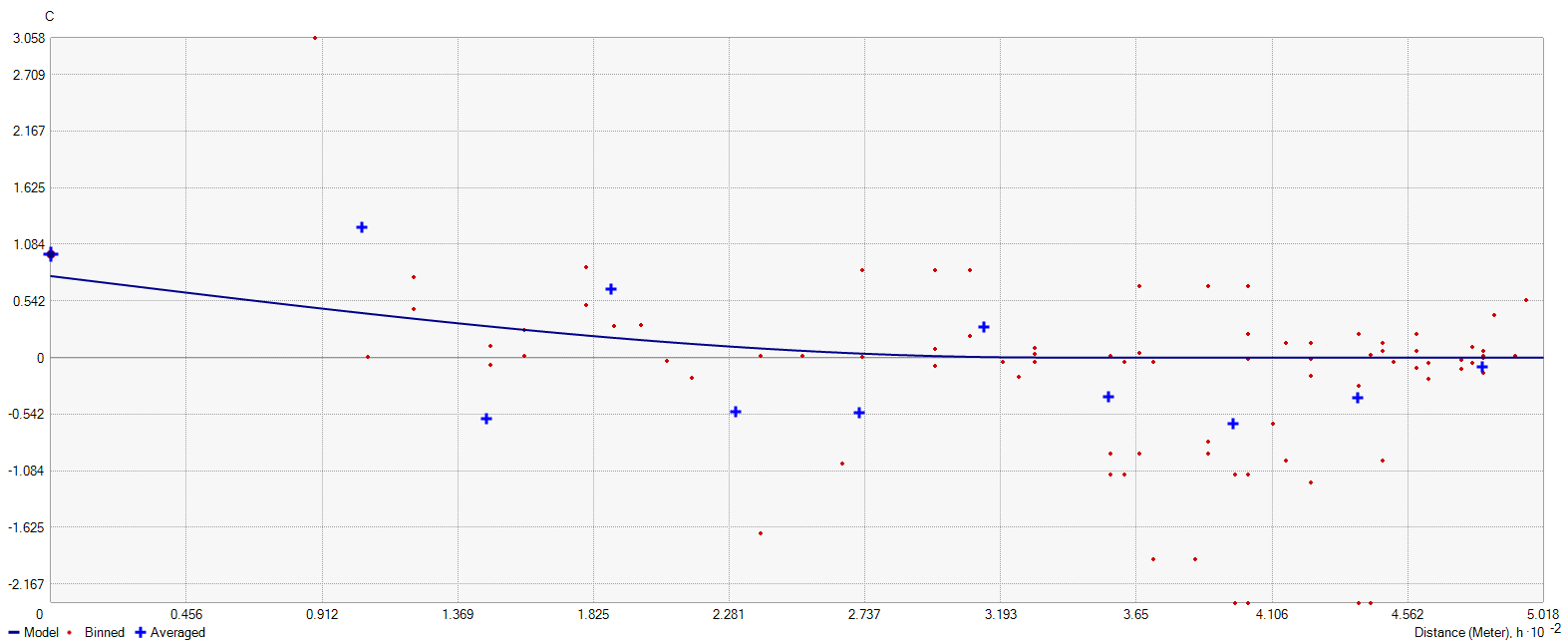


**Cu**: best fit model for Cu (SK with all)


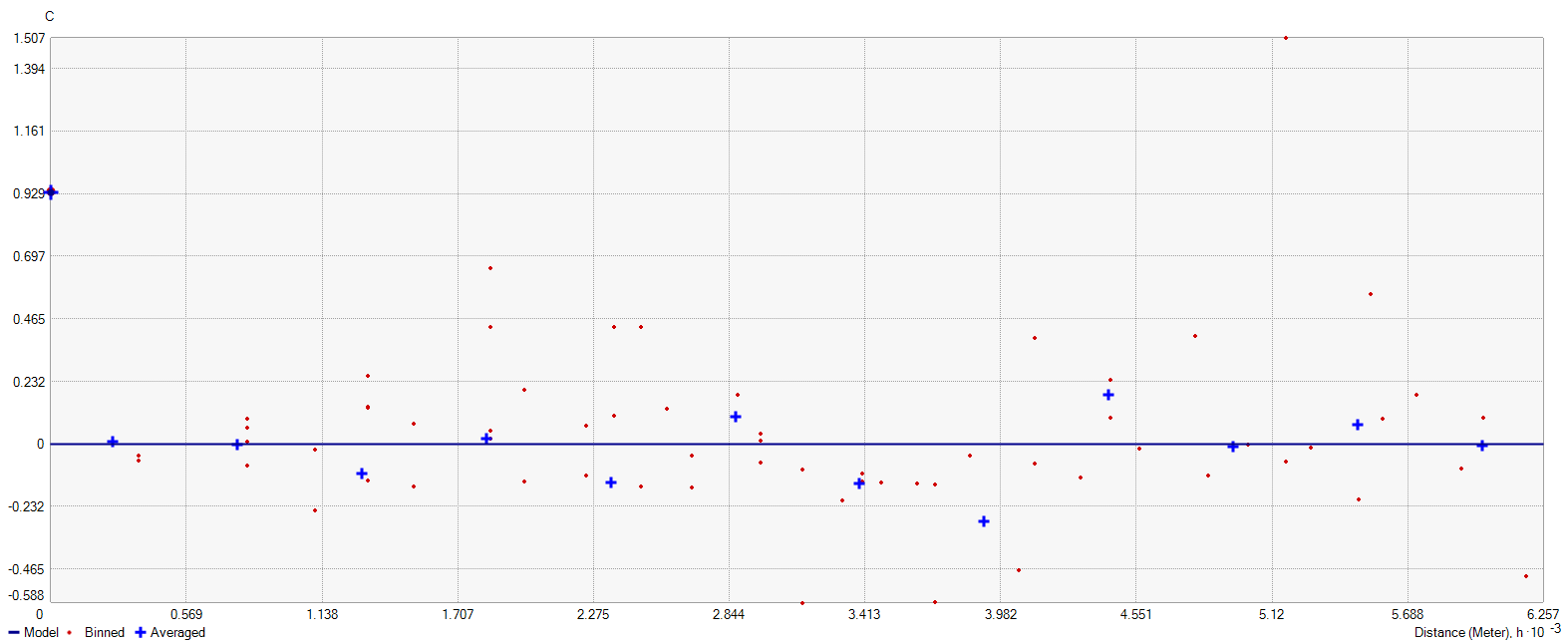


**Zn**: best fit model for Zn (SK with Circular)


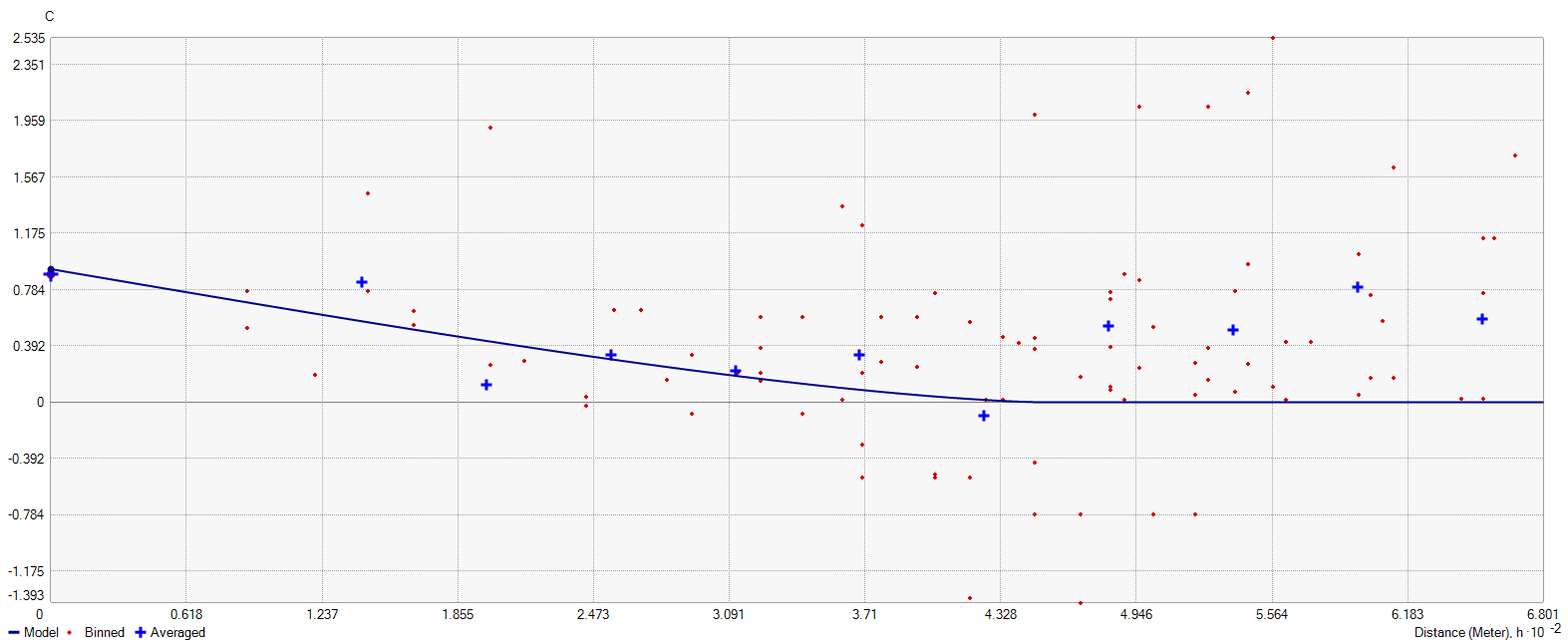


**Fe**: best fit model for Fe (SK with Gaussian)
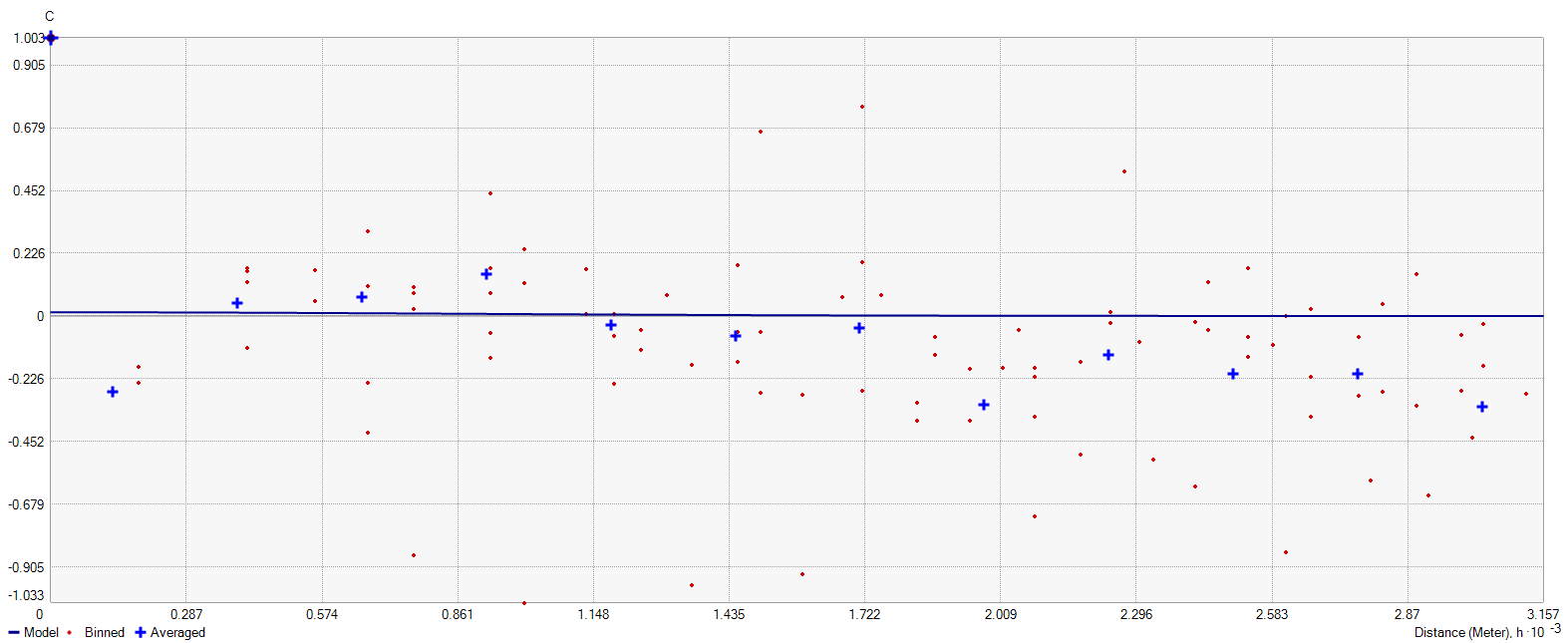


**Mn**: best fit model for Mn (SK with all)
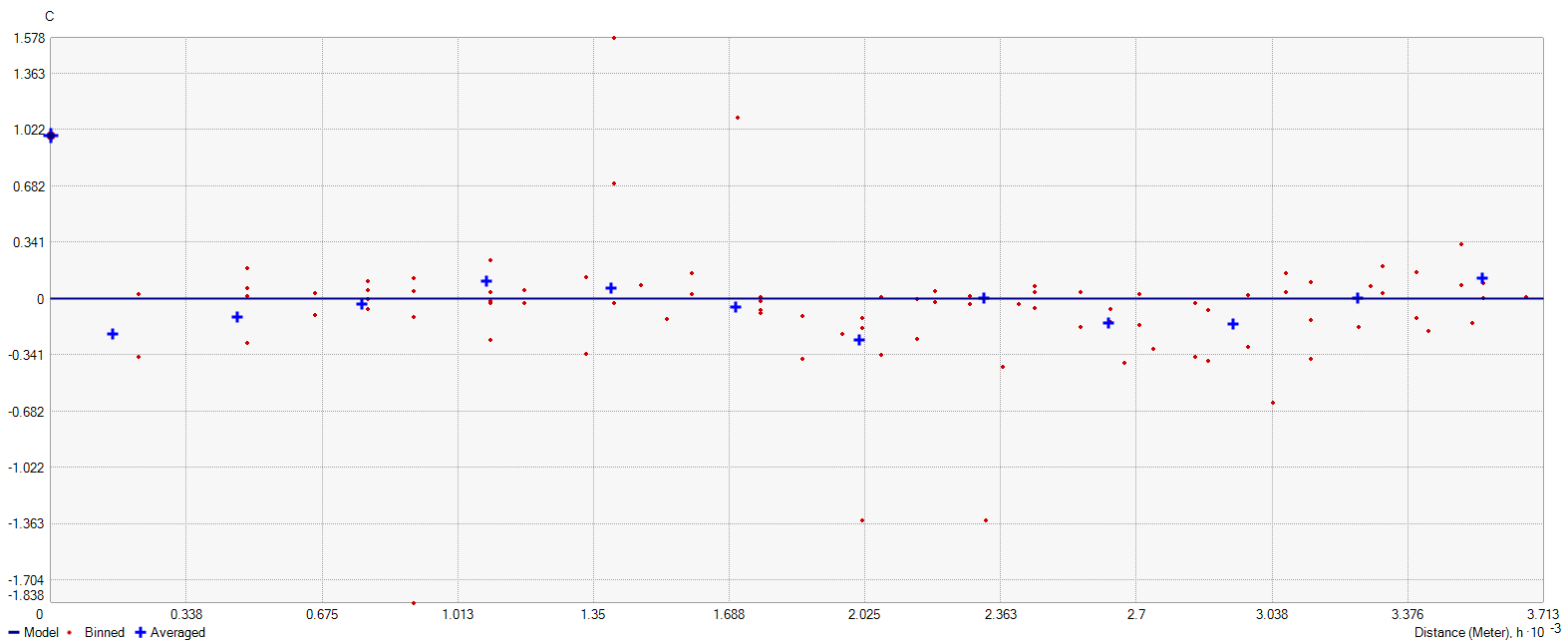


**B**: best fit model for B (SK with all)
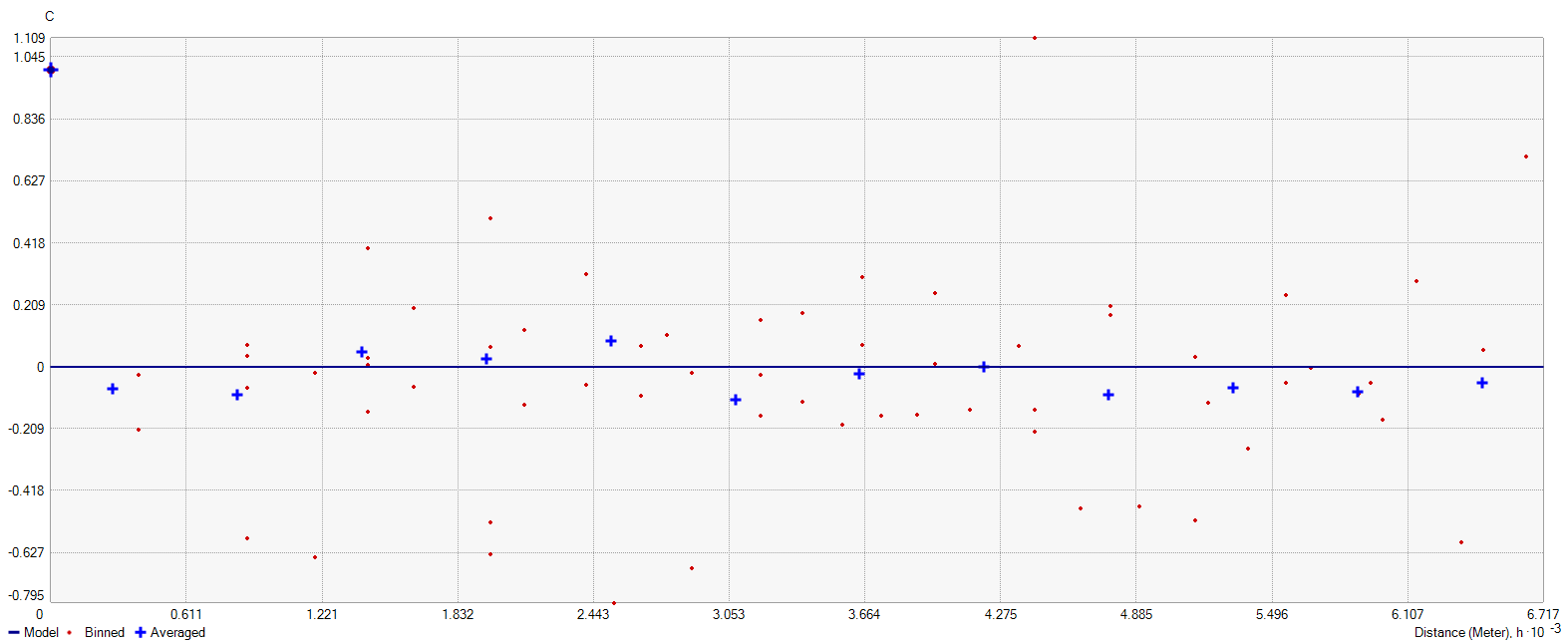


**Figure S2** Semi-variogram model fitting for various pedo-chemical parameters for sub-surface soils

**Supplementary Tables**

**Table S1** Location of soil samples collection

| Location Id. | Latitude | Longitude | Depth of soil samples collection (m) | |
| --- | --- | --- | --- | --- |
|  |  |  | Surface soil samples (A) | Sub-surface soil samples (B) |
| 1 | 23° 33' 11.29"N | 87° 12' 25.18999" E | 0 - 0.1 | 0.7 |
| 2 | 23° 33' 5.01998"N | 87° 12' 21.35999"E | 0 - 0.1 | 0.7 |
| 3 | 23° 32' 59.01"N | 87° 12' 17.74001"E | 0 - 0.1 | 0.7 |
| 4 | 23° 33' 21.57001"N | 87° 11' 48.10999"E | 0 - 0.1 | 0.7 |
| 5 | 23° 33' 18.60998"N | 87° 11' 45.48998"E | 0 - 0.1 | 0.7 |
| 6 | 23° 33' 15.41002"N | 87° 11' 42.15998"E | 0 - 0.1 | 0.7 |
| 7 | 23° 33' 30.84001"N | 87° 11' 36.35002"E | 0 - 0.1 | 0.7 |
| 8 | 23° 33' 25.66001"N | 87° 11' 34.55002" E | 0 - 0.1 | 0.7 |
| 9 | 23° 33' 20.22998" N | 87° 11' 32.66002"E | 0 - 0.1 | 0.7 |
| 10 | 23° 33' 31.21999"N | 87° 11' 19.18"E | 0 - 0.1 | 0.7 |
| 11 | 23° 33' 27.13"N | 87° 11' 16.28999" E | 0 - 0.1 | 0.7 |
| 12 | 23° 33' 24.41999"N | 87° 11' 12.41002"E | 0 - 0.1 | 0.7 |
| 13 | 23° 32' 7.44"N | 87° 13' 31.0908"E | 0 - 0.1 | 0.7 |
| 14 | 23° 32' 0.24"N | 87° 13' 24.96"E | 0 - 0.1 | 0.7 |
| 15 | 23° 31' 51.96" N | 87° 13' 18.12" E | 0 - 0.1 | 0.7 |
| 16 | 23° 31' 36.12" N | 87° 14' 8.88" E | 0 - 0.1 | 0.7 |
| 17 | 23° 31' 31.44" N | 87° 14' 4.92" E | 0 - 0.1 | 0.7 |
| 18 | 23° 31' 25.68" N | 87° 14' 0.24" E | 0 - 0.1 | 0.7 |
| 19 | 23° 32' 52.7244" N | 87° 12' 51.7824" E | 0 - 0.1 | 0.7 |
| 20 | 23° 32' 45.0852" N | 87° 12' 45.3816" E | 0 - 0.1 | 0.7 |
| 21 | 23° 32' 36.0924" N | 87° 12' 37.314" E | 0 - 0.1 | 0.7 |
| 22 | 23° 32' 26.6568" N | 87° 13' 6.6288" E | 0 - 0.1 | 0.7 |
| 23 | 23° 32' 21.0588" N | 87° 13' 2.6868" E | 0 - 0.1 | 0.7 |
| 24 | 23° 32' 13.56" N | 87° 12' 56.9556" E | 0 - 0.1 | 0.7 |
| 25 | 23° 31' 53.5584" N | 87° 13' 51.654" E | 0 - 0.1 | 0.7 |
| 26 | 23° 31' 45.6636" N | 87° 13' 45.0804" E | 0 - 0.1 | 0.7 |
| 27 | 23° 31' 38.7444" N | 87° 13' 38.9712" E | 0 - 0.1 | 0.7 |
| 28 | 23° 33' 35.4888" N | 87° 11' 1.3236" E | 0 - 0.1 | 0.7 |
| 29 | 23° 33' 31.8348" N | 87° 10' 59.7576" E | 0 - 0.1 | 0.7 |
| 30 | 23° 33' 28.116" N | 87° 10' 58.3212" E | 0 - 0.1 | 0.7 |

**Table S2** Computed PI and PLI values of the soil samples

| Location Id. | PI | | PLI | |
| --- | --- | --- | --- | --- |
|  | A | B | A | B |
| 1 | 0.041636 | 0.053592 | 0.013500 | 0.011700 |
| 2 | 0.07203 | 0.043214 | 0.012383 | 0.011293 |
| 3 | 0.084198 | 0.062159 | 0.019148 | 0.015118 |
| 4 | 0.043676 | 0.037171 | 0.015104 | 0.012950 |
| 5 | 0.05865 | 0.0364 | 0.016831 | 0.013639 |
| 6 | 0.092763 | 0.073426 | 0.016090 | 0.013261 |
| 7 | 0.073314 | 0.064733 | 0.020363 | 0.017054 |
| 8 | 0.041494 | 0.046477 | 0.015138 | 0.014552 |
| 9 | 0.088034 | 0.069649 | 0.017217 | 0.012664 |
| 10 | 0.051204 | 0.04385 | 0.012323 | 0.011513 |
| 11 | 0.064126 | 0.04297 | 0.017322 | 0.014970 |
| 12 | 0.039938 | 0.033316 | 0.010771 | 0.010495 |
| 13 | 0.076094 | 0.050352 | 0.016186 | 0.012858 |
| 14 | 0.043898 | 0.031073 | 0.010852 | 0.009211 |
| 15 | 0.05764 | 0.045235 | 0.011003 | 0.009656 |
| 16 | 0.05669 | 0.035953 | 0.012744 | 0.010583 |
| 17 | 0.080714 | 0.064535 | 0.014718 | 0.012705 |
| 18 | 0.060758 | 0.052366 | 0.014736 | 0.014389 |

**Table S3** Computed RI and Eir values of the soil samples

| Location Id. | Cu | | Zn | | Mn | | RI | |
| --- | --- | --- | --- | --- | --- | --- | --- | --- |
|  | Eir | | | | | | A | B |
|  | A | B | A | B | A | B |  |  |
| 1 | 0.066372 | 0.1 | 0.038462 | 0.020362 | 0.052632 | 0.070478 | 0.157465 | 0.19084 |
| 2 | 0.055752 | 0.093805 | 0.013575 | 0.015837 | 0.097081 | 0.056603 | 0.166408 | 0.166245 |
| 3 | 0.075221 | 0.061062 | 0.054299 | 0.052036 | 0.110144 | 0.080048 | 0.239663 | 0.193146 |
| 4 | 0.171681 | 0.138938 | 0.039819 | 0.031674 | 0.052871 | 0.045455 | 0.264371 | 0.216067 |
| 5 | 0.143363 | 0.161062 | 0.033937 | 0.042986 | 0.075311 | 0.037416 | 0.25261 | 0.241465 |
| 6 | 0.079646 | 0.075221 | 0.024887 | 0.027149 | 0.124402 | 0.097703 | 0.228935 | 0.200074 |
| 7 | 0.114159 | 0.088496 | 0.056561 | 0.080543 | 0.094019 | 0.075024 | 0.26474 | 0.244062 |
| 8 | 0.09646 | 0.116814 | 0.042986 | 0.058371 | 0.051196 | 0.038278 | 0.190643 | 0.213463 |
| 9 | 0.086726 | 0.095575 | 0.054299 | 0.033937 | 0.115311 | 0.091579 | 0.256335 | 0.221091 |
| 10 | 0.075221 | 0.076106 | 0.027149 | 0.031674 | 0.066986 | 0.056316 | 0.169356 | 0.164096 |
| 11 | 0.118584 | 0.136283 | 0.037104 | 0.047511 | 0.08311 | 0.05177 | 0.238798 | 0.235565 |
| 12 | 0.084956 | 0.088496 | 0.029412 | 0.040724 | 0.050909 | 0.035885 | 0.165277 | 0.165105 |
| 13 | 0.06531 | 0.097345 | 0.031674 | 0.024887 | 0.1011 | 0.065598 | 0.198084 | 0.18783 |
| 14 | 0.073451 | 0.081416 | 0.022624 | 0.024887 | 0.057321 | 0.039043 | 0.153396 | 0.145346 |
| 15 | 0.050442 | 0.066372 | 0.020362 | 0.017647 | 0.07689 | 0.059761 | 0.147694 | 0.14378 |
| 16 | 0.110619 | 0.123894 | 0.024887 | 0.020362 | 0.074163 | 0.045455 | 0.209669 | 0.18971 |
| 17 | 0.063717 | 0.075221 | 0.027149 | 0.018552 | 0.107895 | 0.086124 | 0.198761 | 0.179898 |
| 18 | 0.132743 | 0.145133 | 0.029412 | 0.039367 | 0.078947 | 0.065933 | 0.241102 | 0.250432 |

**Table S4** Computed Pi values of the soil samples

| Location A | | |  | Location B | | |
| --- | --- | --- | --- | --- | --- | --- |
| Site | Pi | Rank |  | Site | Pi | Rank |
| 1 | 0.795606 | 3 |  | 1 | 0.973018 | 15 |
| 2 | 0.797773 | 2 |  | 2 | 0.985276 | 1 |
| 3 | 0.672236 | 19 |  | 3 | 0.963849 | 20 |
| 4 | 0.290104 | 30 |  | 4 | 0.968054 | 18 |
| 5 | 0.533978 | 25 |  | 5 | 0.955474 | 27 |
| 6 | 0.693662 | 15 |  | 6 | 0.967548 | 19 |
| 7 | 0.610722 | 22 |  | 7 | 0.936488 | 29 |
| 8 | 0.71678 | 12 |  | 8 | 0.957908 | 25 |
| 9 | 0.454622 | 27 |  | 9 | 0.037546 | 30 |
| 10 | 0.705239 | 13 |  | 10 | 0.974933 | 14 |
| 11 | 0.631756 | 20 |  | 11 | 0.95948 | 24 |
| 12 | 0.463835 | 26 |  | 12 | 0.95632 | 26 |
| 13 | 0.756942 | 6 |  | 13 | 0.979916 | 4 |
| 14 | 0.691085 | 16 |  | 14 | 0.970932 | 16 |
| 15 | 0.80885 | 1 |  | 15 | 0.98213 | 2 |
| 16 | 0.416356 | 28 |  | 16 | 0.968253 | 17 |
| 17 | 0.741527 | 11 |  | 17 | 0.978285 | 6 |
| 18 | 0.392371 | 29 |  | 18 | 0.949771 | 28 |
| 19 | 0.748271 | 8 |  | 19 | 0.975527 | 11 |
| 20 | 0.743672 | 9 |  | 20 | 0.975379 | 13 |
| 21 | 0.741582 | 10 |  | 21 | 0.975432 | 12 |
| 22 | 0.764114 | 5 |  | 22 | 0.978857 | 5 |
| 23 | 0.773603 | 4 |  | 23 | 0.977714 | 7 |
| 24 | 0.752564 | 7 |  | 24 | 0.980077 | 3 |
| 25 | 0.700757 | 14 |  | 25 | 0.976875 | 8 |
| 26 | 0.683742 | 17 |  | 26 | 0.976008 | 10 |
| 27 | 0.680957 | 18 |  | 27 | 0.976291 | 9 |
| 28 | 0.62142 | 21 |  | 28 | 0.962574 | 23 |
| 29 | 0.603447 | 24 |  | 29 | 0.963789 | 21 |
| 30 | 0.608663 | 23 |  | 30 | 0.963212 | 22 |

**Table S5** Comparison of interpolation models of pedo-chemical parameters

| **Parameter** | **Model** | | **Best fit model** | | **ME** | | **RMSE** | | **MSE** | | **RMSSE** | | **ASE** | |
| --- | --- | --- | --- | --- | --- | --- | --- | --- | --- | --- | --- | --- | --- | --- |
|  | **A** | **B** | **A** | **B** | **A** | **B** | **A** | **B** | **A** | **B** | **A** | **B** | **A** | **B** |
| pH | SK | SK | All | All | -0.02 | 0.07 | 0.58 | **0.51** | -0.04 | 0.13 | 0.99 | 0.87 | 0.59 | 0.58 |
|  | OK | OK | Circular | All | -0.01 | -0.02 | **0.58** | 0.53 | -0.01 | -0.03 | 0.94 | 0.96 | 0.62 | 0.56 |
|  | IDW | IDW | P1 | P1 | -0.01 | -0.04 | 0.64 | 0.61 | - | - | - | - | - | - |
| EC | SK | SK | Spherical | Gaussian | 0.00 | 0.00 | 0.14 | **0.08** | 0.02 | -0.02 | 0.90 | 1.17 | 0.15 | 0.07 |
|  | OK | OK | Circular | All | -0.01 | 0.00 | **0.14** | 0.08 | -0.03 | 0.00 | 0.90 | 0.95 | 0.15 | 0.09 |
|  | IDW | IDW | P1 | P1 | 0.00 | 0.00 | 0.16 | 0.09 | - | - | - | - | - | - |
| OC | SK | SK | All | Gaussian | 0.00 | 0.00 | **0.07** | **0.07** | 0.00 | 0.02 | 0.94 | 0.94 | 0.07 | 0.08 |
|  | OK | OK | All | Circular | 0.00 | 0.01 | 0.07 | 0.08 | 0.00 | 0.06 | 0.99 | 0.69 | 0.07 | 0.12 |
|  | IDW | IDW | P1 | P2 | 0.00 | 0.01 | 0.08 | 0.09 | - | - | - | - | - | - |
| CEC | SK | SK | All | Exponential | 0.00 | 0.04 | **0.81** | 1.45 | 0.00 | 0.02 | 1.00 | 0.94 | 0.81 | 1.67 |
|  | OK | OK | All | Circular | 0.00 | 0.01 | 0.84 | 1.48 | 0.00 | 0.01 | 0.98 | 0.77 | 0.86 | 2.06 |
|  | IDW | IDW | P1 | P2 | -0.02 | -0.01 | 0.96 | 1.54 | - | - | - | - | - | - |
| Sand | SK | SK | Gaussian | Circular | 0.26 | -0.34 | **6.15** | 5.87 | 0.03 | 0.05 | 0.95 | 1.27 | 6.46 | 6.86 |
|  | OK | OK | All | Gaussian | 0.16 | 0.15 | 6.16 | **5.59** | 0.02 | 0.04 | 0.96 | 0.95 | 6.41 | 5.86 |
|  | IDW | IDW | P1 | P2 | -0.05 | -0.39 | 6.52 | 6.00 | - | - | - | - | - | - |
| Silt | SK | SK |  | Exponential | -0.14 | 0.15 | 5.54 | 6.37 | -0.01 | -0.08 | 0.95 | 1.18 | 5.65 | 6.80 |
|  | OK | OK | Exponential/Gaussian | Gaussian | -0.32 | -0.02 | **5.47** | **5.69** | -0.06 | -0.02 | 1.03 | 1.00 | 5.30 | 5.52 |
|  | IDW | IDW | P1 | P2 | 0.03 | 0.42 | 5.76 | 6.32 | - | - | - | - | - | - |
| Clay | SK | SK | Gaussian | Circular | -0.02 | 0.02 | **2.02** | 1.48 | -0.01 | 0.02 | 1.00 | 0.94 | 2.03 | 1.58 |
|  | OK | OK | All | Gaussian | -0.05 | 0.00 | 2.10 | **1.46** | -0.02 | 0.00 | 0.94 | 0.96 | 2.22 | 1.58 |
|  | IDW | IDW | P1 | P2 | 0.01 | 0.00 | 2.21 | 1.55 | - | - | - | - | - | - |
| Moisture | SK | SK |  | Spherical | -0.14 | -0.13 | **2.40** | **2.05** | -0.07 | -0.02 | 1.24 | 1.08 | 1.94 | 1.58 |
|  | OK | OK | All | Circular | -0.09 | 0.02 | 2.52 | 2.30 | -0.04 | 0.00 | 1.00 | 0.77 | 2.51 | 3.04 |
|  | IDW | IDW | P1 | P1 | 0.03 | 0.05 | 2.53 | 2.33 | - | - | - | - | - | - |
| Cu | SK | SK | Gaussian | All | 0.04 | -0.01 | **0.26** | 0.25 | 0.10 | -0.03 | 1.06 | 1.02 | 0.25 | 0.25 |
|  | OK | OK | Spherical | All | 0.00 | -0.01 | 0.27 | 0.27 | -0.02 | -0.02 | 0.94 | 0.96 | 0.29 | 0.28 |
|  | IDW | IDW | P1 | P1 | 0.00 | 0.01 | 0.27 | 0.28 | - | - | - | - | - | - |
| Zn | SK | SK | Gaussian | Circular | 0.00 | 0.02 | 0.20 | **0.24** | 0.01 | 0.06 | 0.97 | 1.02 | 0.20 | 0.25 |
|  | OK | OK | All | Circular | 0.00 | -0.01 | **0.20** | 0.25 | 0.01 | -0.02 | 0.95 | 0.90 | 0.21 | 0.30 |
|  | IDW | IDW | P1 | P1 | 0.00 | 0.00 | 0.21 | 0.26 | - | - | - | - | - | - |
| Fe | SK | SK | Gaussian | Gaussian | 0.09 | -0.01 | 8.47 | **4.76** | 0.01 | 0.00 | 0.98 | 1.00 | 8.65 | 4.77 |
|  | OK | OK | Gaussian | All | 0.15 | -0.06 | **8.34** | 4.89 | 0.01 | -0.01 | 0.93 | 0.98 | 9.01 | 4.92 |
|  | IDW | IDW | P1 | P1 | 0.01 | 0.00 | 8.78 | 5.28 | - | - | - | - | - | - |
| Mn | SK | SK | All | All | 0.00 | 0.00 | **3.85** | **3.12** | 0.00 | 0.00 | 0.99 | 1.01 | 3.89 | 3.10 |
|  | OK | OK | All | All | 0.10 | 0.01 | 4.04 | 3.33 | 0.02 | 0.00 | 0.99 | 1.01 | 4.08 | 3.30 |
|  | IDW | IDW | P1 | P1 | -0.02 | -0.10 | 4.37 | 3.54 | - | - | - | - | - | - |
| B | SK | SK | All | All | 0.00 | 0.00 | **0.07** | **0.07** | 0.00 | 0.00 | 0.99 | 1.00 | 0.07 | 0.07 |
|  | OK | OK | All | All | 0.00 | 0.00 | 0.08 | 0.07 | -0.02 | -0.01 | 1.00 | 1.02 | 0.08 | 0.07 |
|  | IDW | IDW | P1 | P1 | 0.00 | 0.00 | 0.09 | 0.08 | - | - | - | - | - | - |
